# UBE2T promotes β‐catenin nuclear translocation in hepatocellular carcinoma through MAPK/ERK‐dependent activation

**DOI:** 10.1002/1878-0261.13111

**Published:** 2022-03-16

**Authors:** Elisavet Lioulia, Panagiotis Mokos, Emmanuel Panteris, Dimitra Dafou

**Affiliations:** ^1^ Department of Genetics, Development and Molecular Biology School of Biology Aristotle University of Thessaloniki Greece; ^2^ Department of Botany School of Biology Aristotle University of Thessaloniki Greece

**Keywords:** E‐cadherin, EMT, HCC, MAPK/ERK, UBE2T, β‐catenin

## Abstract

Ubiquitin‐conjugating enzyme E2T (UBE2T) has been implicated in many types of cancer including hepatocellular carcinoma (HCC). Epithelial–mesenchymal transition (EMT) process plays a fundamental role during tumor metastasis and progression. However, the molecular mechanisms underlying EMT in HCC in accordance with UBE2T still remain unknown. In this study, we showed that UBE2T overexpression augmented the oncogenic properties and specifically EMT in HCC cell lines, while its silencing attenuated them. UBE2T affected the activation of EMT‐associated signaling pathways: MAPK/ERK, AKT/mTOR, and Wnt/β‐catenin. In addition, we revealed that the epithelial protein complex of E‐cadherin/β‐catenin, a vital regulator of signal transduction in tumor initiation and progression, was totally disrupted at the cell membrane. In particular, we observed that UBE2T overexpression led to E‐cadherin loss accompanied by a simultaneous elevation of both cytoplasmic and nuclear β‐catenin, while its silencing resulted in a strong E‐cadherin turnover at the cell membrane. Interestingly, chemical inhibition of the MAPK/ERK, AKT/mTOR, and Wnt/β‐catenin signaling pathways demonstrated that the nuclear translocation of β‐catenin and subsequent EMT was enhanced mainly by MAPK/ERK. Collectively, our findings demonstrate the UBE2T/MAPK‐ERK/β‐catenin axis as a critical regulator of cell state transition and EMT in HCC.

## Graphical abstract

Hepatocellular carcinoma (HCC) is the third leading cause of cancer deaths worldwide. In this study, the authors investigated the role of ubiquitin‐conjugating enzyme E2T (UBE2T) in epithelial–mesenchymal transition (EMT) in HCC. UBE2T promoted EMT via E‐cadherin/β‐catenin disruption at the cell membrane and β‐catenin nuclear translocation through MAPK‐ERK‐dependent activation. Therefore, the UBE2T/MAPK‐ERK/β‐catenin axis may serve as a promising target for HCC treatment.

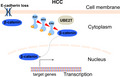

**Abbreviations**
AKT: protein kinase B
EMT: epithelial–mesenchymal transition
ERK: extracellular signal‐regulated kinase
HBV: hepatitis B virus
HCC: hepatocellular carcinoma
HCV: hepatitis C virus
MAPK: mitogen‐activated protein kinase
MET: mesenchymal–epithelial transition
mTOR: mammalian target of rapamycin
siRNA: small interfering RNA
UBE2T: ubiquitin‐conjugating enzyme E2T
UP: ubiquitin proteasome
Wnt: wingless integrated

## 1 Introduction

Hepatocellular carcinoma (HCC) is one of the most malignant tumors worldwide and the third leading cause of cancer deaths. The incidence of HCC often occurs in the background of cirrhotic liver, initiating from chronic infection with hepatitis B (HBV), hepatitis C (HCV), or systematic alcohol consumption among the major risk factors [1]. Despite aggressive treatment regimes, including surgery, combined radio, and chemotherapy, HCC patients will still die due to tumor recurrence and metastasis with the death rates increasing by ~ 2–3% per year [2]. The molecular mechanisms underlying HCC development still remain poorly understood, although several oncogenes and tumor suppressor genes have been implicated in the initiation and progression of HCC [3, 4, 5].

Epithelial–mesenchymal transition (EMT), a cellular process in which epithelial cells acquire mesenchymal features, has been related to liver cancer progression [6, 7].

PI3K/AKT/mTOR, MAPK/ERK, Wnt/β‐catenin, and the ubiquitin proteasome (UP) represent the major signal transduction pathways implicated in EMT and liver cancer progression [8, 9, 10, 11, 12, 13, 14]. A key signaling modulator, the E‐cadherin/β‐catenin protein complex located normally at cell–cell contacts, plays a crucial role in epithelial homeostasis, EMT, and tumor progression [15]. Loss of E‐cadherin at the membrane and subsequent β‐catenin release into the nucleus results in different cell shapes, activation of EMT signal transduction pathways, and tumor metastasis [16], while E‐cadherin overexpression in some tumor cell lines decreased EMT [17, 18, 19, 20].

Many signal transducers of EMT, including the E‐cadherin/β‐catenin protein complex, are regulated by the ubiquitin signaling [21, 22]. The UP system regulates an array of cellular distinct functions, such as RNA biogenesis, processing, and protein modification, indicating its significant potential for novel therapeutic strategies in cancer [23, 24].

Ubiquitin‐conjugating enzyme E2T (UBE2T) is a member of the E2 family in the UP pathway. Ubiquitin signaling is typically performed by E1, E2, and E3 enzymes which catalyze the activation, conjugation, and ligation processes, respectively [25]. In particular, UBE2T has been related to many cellular functions such as DNA damage, genome instability, proliferation, and differentiation [26, 27, 28]. Also, it has been characterized as an oncogene in many cancer types such as nasopharyngeal, renal, and HCC [29, 30, 31, 32, 33].

The present study shows the impact of UBE2T expression on the oncogenic properties in HCC and specifically in the EMT process. EMT induction was noticed after UBE2T overexpression, resulting in a significant E‐cadherin loss and a MAPK/ERK‐dependent β‐catenin nuclear accumulation. By unraveling the molecular mechanisms of β‐catenin translocation from plasma membrane to the nucleus and E‐cadherin relocalization at the cell membrane, in accordance with UBE2T expression levels, we could identify novel potential therapeutic strategies in HCC.

## 2 Materials and methods

### 2.1 Cell culture, transfections, and treatments

The HCC cell lines HepG2/Huh7 (ATCC, Manassas,VA, USA) were cultured in DMEM (Dulbecco's modified Eagle medium, #41966‐029), supplemented with 10% heat‐inactivated FBS (#10270‐106), 1% NEAA (nonessential amino acids, #11140‐035), and 1% GlutaMAX (#35050‐061), all from Thermo Fischer Scientific (Waltham, MA, USA). For UBE2T overexpression experiments, 1 × 10^6^ cells were seeded and the following day were transfected with the KAR32 MSCV_C_HA_FLAG UBE2T expression plasmid and empty vector control, using Lipofectamine 2000 (#11668‐019; Thermo Fischer Scientific), as per manufacturer's instructions. UBE2T transcriptional knockdown was achieved by FlexiTube^®^ of two preselected siRNAs (1 nmol, Hs_UBE2T_1, #SI03106355 & Hs_UBE2T_2, #SI04439421) compared with the negative control siRNA (All Stars Negative, #1027280), all sequences from QIAGEN (Hilden, Germany) using Lipofectamine RNAiMAX (#13778‐030; Thermo Fischer Scientific), as per manufacturer's instructions. Cells were further analyzed 48 h post‐transfection. Stable UBE2T overexpression monoclonal cell lines were generated by similar transfection conditions followed by puromycin selection (#P8833; Sigma‐Aldrich, St. Louis, MO, USA, 1 μg·mL^−1^). As for the inhibitor treatment, inhibition of: AKT was achieved with MK‐2206 (#HY‐10358, 5 μm), Wnt with IWP‐2 (#HY‐13912, 5 μm), Wnt/β‐catenin with IWR‐1 (#HY‐12238, 5 μm) and ERK with SCH‐772984 (#HY‐50846, 0.1 μm), all from MedChemExpress (Stockholm, Sweden), with matching DMSO (Dimethyl Sulfoxide; Sigma‐Aldrich, #D5879) solvent controls. As for TOPflash/FOPflash luciferase assay, we used the following plasmids: KAR32 MSCV_C_HA_FLAG UBE2T expression plasmid and an empty vector control, TOPflash, FOPflash, and β‐galactosidase plasmids. The luciferase‐reporter constructs have been described previously [34]. Cells were cultured at 37 °C into a humidified incubator with 5% CO_2_, used for 3–5 passages, and tested regularly for mycoplasma contamination.

### 2.2 Soft agar colony‐formation assay

The soft agar colony‐formation assay was performed in 6‐well plates. 5 × 10^3^ cells were suspended in complete medium mixed with a 0.33% top agar layer (#A5431; Sigma‐Aldrich) and plated on a 0.66% bottom agar layer mixed with complete medium. After 4 weeks of undisturbed growth, visible colonies in five randomly selected fields were counted and captured with a Zeiss Axiovert 40C Inverted Microscope (Carl Zeiss, Berlin, Germany).

### 2.3 Cell migration assay

Cells (2 × 10^4^) were seeded into Ibidi silicone inserts with a defined cell‐free gap, and the next day inserts were removed. The cell‐free width was captured at 24 and 48h with a Zeiss Axiovert 40C Inverted Microscope, and images were analyzed by imagej (https://imagej.nih.gov/ij/).

### 2.4 Cell invasion assay

Cells (2 × 10^5^) were seeded into the upper compartment of the invasion chamber of the Chemicon Cell Invasion Assay Kit (#ECM550; EMD Millipore Corporation, Burlington, MA, USA) in serum‐free medium. Medium with 10% FBS was added to the lower compartment. After 48‐h incubation, cells on the upper side were washed and removed, while invaded cells on the lower side were stained, counted, and captured with Zeiss Axiovert 40C Inverted Microscope.

### 2.5 Cell proliferation assay

Cells (5 × 10^3^) were seeded into a 96‐well plate, and one day postseeding, alamar blue (#Y00‐010; Thermo Fischer Scientific) was added, as per manufacturer's instructions. 570/630 nm absorbance was measured every 24 h for a total period of 4 days.

### 2.6 Cell viability assay

Cells were plated on sterile glass coverslips. After 48 h, we stained and distinguished viable cells with calcein AM (1 μm) and dead cells with EthD‐1(Ethidium Homodimer‐1, 2 μm), using the LIVE/DEAD^®^ Viability/Cytotoxicity Kit (#L‐3224; Invitrogen, Waltham, MA, USA). Following 40‐min incubation into a humidified incubator and two PBS (phosphate‐buffered saline, #10010‐023; Thermo Fischer Scientific) washes, confocal images were captured by the inverted microscope Zeiss Axio Observer Z1, equipped with the laser scanning unit LSM 780. Digital images were acquired with zen2011 software (Carl Zeiss, Berlin, Germany).

### 2.7 Cell cycle analysis

Cells (1 × 10^6^) were harvested 48 h after treatment, washed twice with PBS, and centrifuged at 300 *g* for 5 min. Regarding DNA content measurement, supernatant was discarded and ice‐cold 70% ethanol was added to fix cells at −20 °C (1 h). Following centrifugation and PBS washes, 400 μL of staining solution containing RNase A (#R6513; Sigma‐Aldrich) and propidium iodide (#P3566; Invitrogen) was added for 15 min at room temperature in the dark. DNA content was quantified with a BD FACSCalibur™ cell analyzer (BD Biosciences, Franklin Lakes, NJ, USA). Regarding propidium iodide/Annexin V staining protocol cell pellet was resuspended in 500 μL of 1× Binding Buffer containing propidium iodide (#P3566; Invitrogen) and Annexin V (#A13199; Invitrogen), while incubated for 20 min in the dark. Etoposide (#E1383, Sigma‐Aldrich, 200 μm) was used as an apoptosis inducer‐positive control.

### 2.8 Immunoblotting

Cells were washed with PBS and resuspended in lysis buffer (1% NP‐40, 0.1% SDS, 50 mm Tris/HCl pH: 8, 150 mm NaCl, 5 mm EDTA pH:8, 1 mm DTT, 2 mm EGTA, 0.5% sodium deoxycholate, 50 μm leupeptin) supplemented with phosphatase inhibitor (#A32957; Thermo Fischer Scientific). Protein lysates were quantified by Bradford assay (Bio‐Rad, #500‐0006, Hercules, CA, USA), and equal amounts (20–100 μg) were separated by SDS/PAGE electrophoresis and transferred to PVDF membrane (#IPVH00010; Merck Millipore, Burlington, MA, USA). The membrane was blocked for non‐phosphorylated proteins (5% Milk, 0.5% BSA: bovine serum albumin in 1× PBS/0.1% Tween‐20) and for phosphorylated (5% BSA in 1× TBS/0.1% Tween‐20) for 1 h at room temperature. Then, the membrane was incubated with primary antibodies (Table S1) for 1 h at room temperature or overnight (4 °C). Finally, the membrane was incubated with secondary antibodies (Table S1) conjugated with horseradish peroxidase for 1 h at room temperature. Bands were visualized by Pierce™ ECL Plus Substrate (#A32957; Thermo Fischer Scientific) with a Typhoon FLA 7000 for quantitative phosphorimaging. Blot images were quantified using gelquant.net software (http://gelquant.net/).

### 2.9 Subcellular fractionation

Cells were washed with PBS, resuspended in lysis buffer (10 mm Hepes, 60 mm KCl, 1.5 mm MgCl_2_, 1 mm EDTA, 1 mm EGTA, 0.075% NP‐40, 1 mm DTT, 50 μm leupeptin), and centrifuged at 700 *g* for 5 min. The supernatant contained the membrane/cytoplasm fraction. Following a pellet wash with lysis buffer, the suspension was centrifuged at 700 *g* for 10 min. The supernatant was discarded and the remaining pellet contained the nuclear fraction. Nuclear pellet was further processed with the REAP method [35] for obtaining nuclear protein fraction. Specifically, nuclear pellets were resuspended in 1× Laemmli sample buffer and boiled for 10 min. Extracted proteins were quantified by Bradford assay, and 50 μg of membrane/cytoplasmic and 20 μg of nuclear extract were separated by SDS/PAGE electrophoresis. The next steps were similar to those of Section 2.8. In order to obtain plasma membrane/cytoplasmic fraction, we further processed the previously obtained supernatant (membrane/cytoplasm fraction). After its centrifugation at 10 000 *g* for 10 min, we continued with the supernatant, which contained the plasma membrane and cytoplasmic fraction. Further centrifugation at 100 000 *g* for 1 h separated the supernatant (cytoplasm) from the remaining pellet (plasma membrane).

### 2.10 Immunofluorescence

Cells were plated on sterile glass coverslips for 48 h, fixed with 4% paraformaldehyde (PFA)/PBS for 20 min, and permeabilized in 0.5% Triton X‐100/PBS for 10 min. Cells were blocked for 1 h, incubated overnight (4 °C) with the primary (Table S1) and secondary (Table S1) antibodies for 1 h at room temperature. DAPI (D9542; Sigma‐Aldrich) staining was performed for 10 min. Confocal images were captured by the inverted microscope Zeiss Axio Observer Z1, equipped with the laser scanning unit LSM 780. Digital images were acquired with zen2011 software, and statistical analysis was performed by imagej (https://imagej.nih.gov/ij/).

### 2.11 3D cell cultures

Cells (7 × 10^4^) were seeded into 35‐mm cell culture dishes growing at 1 : 1 ratio of complete medium and Matrigel Growth Factor Reduced (GFR) Basement Membrane Matrix (#354230; BD Biosciences) for 48 h. For the inhibitor treatment, the same concentrations were used as described in Section 2.1. Cells were processed for immunofluorescence, as described in Section 2.10 with slight modifications: increased PFA fixation (2 h) and permeabilization with 0.5% Triton X‐100/PBS (2 h).

### 2.12 TOPflash/FOPflash luciferase assay

Cells (5 × 10^3^) were seeded into a 96‐well plate, and the next day, each well was transfected with a total of 50 ng DNA: 35 ng of KAR32 MSCV_C_HA_FLAG UBE2T expression plasmid or empty vector control, 10 ng of TOPflash or FOPflash, and 5 ng of β‐galactosidase plasmid (control for transfection efficiency) using Lipofectamine 2000. LiCl (#L‐0505; Sigma‐Aldrich, 20 mm) served as an activator of the Wnt/β‐catenin pathway. After 24‐h treatment, we measured luciferase activity using the Luciferase Assay System (#E1501; Promega, Madison, WI, USA), while β‐galactosidase levels were measured with Galacto‐Light Plus™ beta‐Galactosidase Reporter Gene Assay System (#T1007; Applied Biosystems, Waltham, MA, USA), following manufacturer's instructions. Luciferase and β‐galactosidase activity were measured using a luminometer and expressed in light units.

### 2.13 Statistical analysis

Graphs were plotted using graphpad prism Software 6 (GraphPad Software, La Jolla, CA, USA). All values are presented as mean ± SEM from at least three independent experiments with suitable number of technical replicates (at least three). Two‐tailed Student's *t*‐test was used to estimate the statistical significance between two groups. Significance was set up at *P* ≤ 0.05.

## 3 Results

### 3.1 UBE2T increases the growth‐promoting traits of HCC cells

**Fig. 1:**
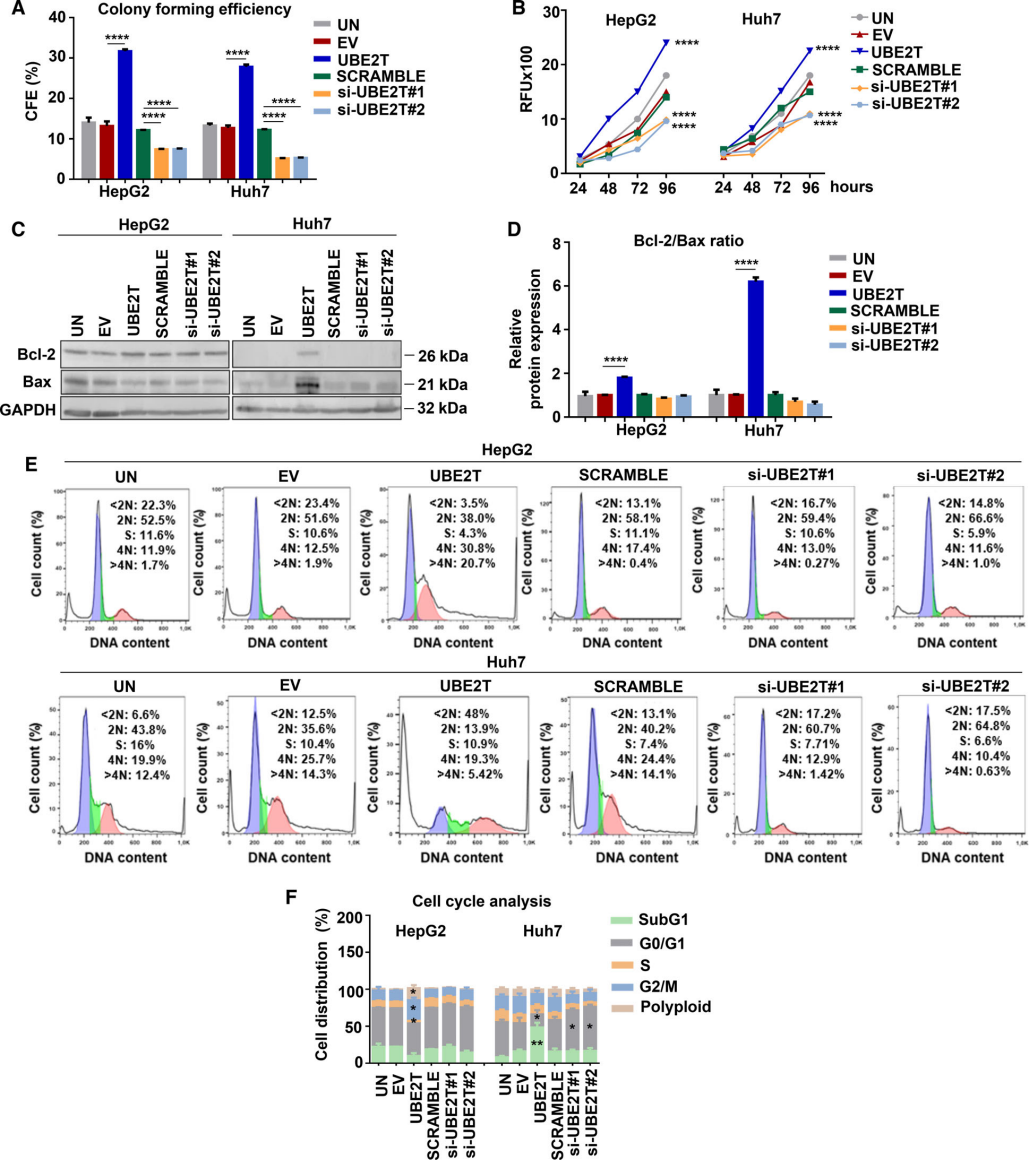
UBE2T increases the growth‐promoting traits of HCC cells. (A) Graph showing the effect of UBE2T expression on soft agar colony formation in HepG2/Huh7. (B) Alamar blue assay was performed to quantify the viability over a period of 96 h. (C) Western blot analysis for the detection of protein levels of Bcl‐2 and Bax. (D) Graph showing the Bcl‐2/Bax ratio from (C). (E) Measurement of DNA content by flow cytometry. (F) Quantification of the percentage of cells in each cell cycle phase from (E). Data shown are the mean ± SEM from *n* = 3 independent experiments, two‐tailed Student's *t*‐test (*P ≤ 0.05, **P ≤ 0.01, ****P ≤ 0.0001). Empty vector (EV)‐transfected cells were used as control for UBE2T ectopic overexpression (UBE2T), while non‐targeting siRNA (SCRAMBLE) as control for UBE2T silencing (si‐UBE2T#1, si‐UBE2T#2). Untransfected cells (UN) were used as negative control. Normalization in western blot was performed to GAPDH.

In order to investigate the potential role of UBE2T in the growth of HCC cells, we overexpressed (UBE2T‐overerxpressing) and silenced (UBE2T‐silencing) its expression for 48 h in HepG2/Huh7 cells (Fig. S1A,B). In colony‐formation assay, which tests the survival of cancer cells [36], we observed enhanced anchorage‐independent growth into semisolid medium of UBE2T overexpressing in contrast to UBE2T‐silencing cells (Fig. 1A and Fig. S1C). The impact of UBE2T expression levels in the proliferation status of cells was investigated by alamar blue assay. Furthermore, we assessed the anti‐apoptotic Bcl‐2/pro‐apoptotic Bax protein expression ratio and calcein‐ethidium staining, which determined cell susceptibility to apoptosis. As shown in Fig. 1B–D, the proliferation and ratio of Bcl‐2/Bax was increased in UBE2T‐overexpressing cells, whereas lower levels were observed in UBE2T silencing. Calcein and ethidium homodimer staining showed an elevated proportion of live (calcein) UBE2T‐overexpressing cells (Fig. S1D).

Cell cycle analysis demonstrated disrupted regulatory mechanisms, which could result in impaired cell division as shown by the observed decreased G0/G1 cell population in UBE2T‐overexpressing cells, whereas UBE2T silencing resulted in its reversion. Decreased G0/G1 cell population led to a G2/M phase arrest and a highly increased number of polyploid cells (*P* ≤ 0.05) in HepG2 UBE2T‐overexpressing cells. In Huh7 UBE2T‐overexpressing cells, an accelerated S‐phase progression was observed with a G2/M phase rapid bypass and apoptosis induction, indicated by the elevated proportion of SubG1 population (*P* ≤ 0.01) (Fig. 1E,F and Fig. S1E). The apoptosis induction was also measured by PI/Annexin V staining (Fig. S1F). Although UBE2T overexpression led to induction of apoptotic signaling in Huh7, the elevated ratio of Bcl‐2/Bax (6‐fold) seems to affect the overall growth of Huh7‐UBE2T cells positively, which is verified by soft agar and alamar blue assay data. Overall, our results indicate the importance of UBE2T in the augmentation of growth promotion of HCC cells.

### 3.2 UBE2T promotes EMT in HCC cells

**Fig. 2:**
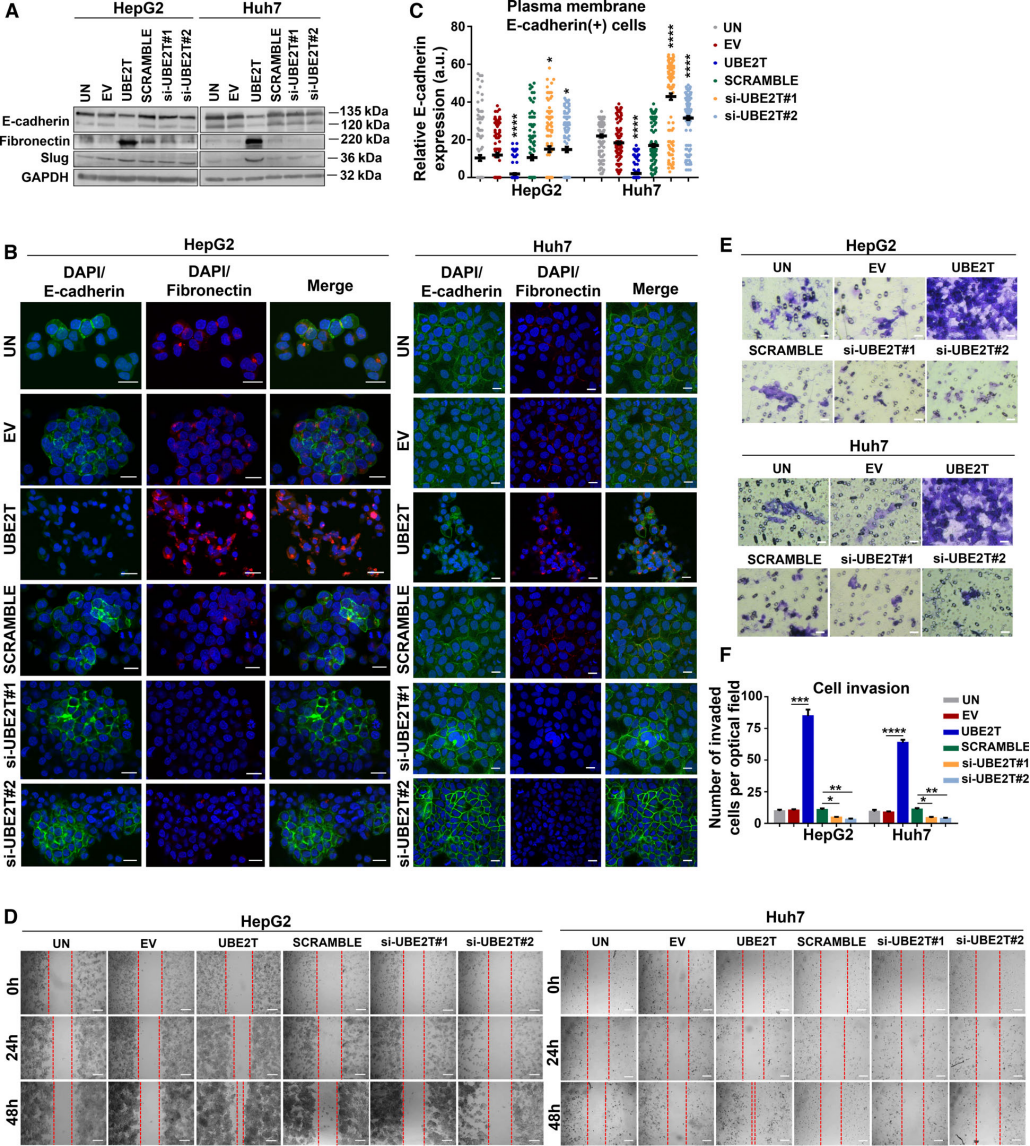
EMT induction in HCC cells by UBE2T overexpression. (A) Western blot analysis of E‐cadherin, fibronectin, and Slug. (B) Maximum projection of serial CLSM sections after immunostaining of UBE2T overexpression or silencing in HepG2/Huh7. Scale bar: 20 μm. E‐cadherin: green, fibronectin: red, DAPI: blue. (C) Scatter plot of intensity values for E‐cadherin at plasma membrane (*n* = 20–70 total cells/optical field) from (B). (D) Scratch assay captured at 24 and 48 h. Scale bar: 200 μm. (E) Invasion assay was performed after 48 h of treatment. Scale bar: 20 μm. (F) Quantification of invasion from (E). Data shown are the mean ± SEM from *n* = 5 independent experiments, two‐tailed Student's *t*‐test (*P ≤ 0.05, **P ≤ 0.01, ***P ≤ 0.001, ****P ≤ 0.0001). Empty vector (EV)‐transfected cells were used as control for UBE2T ectopic overexpression (UBE2T), while nontargeting siRNA (SCRAMBLE) as control for UBE2T silencing (si‐UBE2T#1, si‐UBE2T#2). Untransfected cells (UN) were used as negative control. Normalization in western blot was performed to GAPDH.

UBE2T has been correlated with EMT induction in many types of cancer such as glioblastoma, gastric, and lung cancer [37, 38, 39]. In our study, we assessed EMT induction by protein expression measurement of established molecular markers and observation of cellular property changes [6, 40]. We focused on the expression levels of the epithelial marker E‐cadherin and the mesenchymal marker fibronectin, a component of the extracellular matrix. We observed a clear downregulation in protein levels of E‐cadherin (*P* ≤ 0.0001 for both cell lines) with simultaneous upregulation of fibronectin (*P* ≤ 0.0001 for both cell lines) in UBE2T‐overexpressing cells, compared to control cells (EV). The increased protein levels of the EMT‐activating transcription factor Slug, a repressor of E‐cadherin transcription [41], further validated EMT induction in both cell lines (Fig. 2A, Fig. S2A). Immunofluorescence analysis confirmed that UBE2T‐overexpressing cells adopt a mesenchymal phenotype with increased fibronectin expression in both cell lines and decreased expression of Ε‐cadherin, detected mainly in the cytoplasm (Fig. 2B).

UBE2T silencing reverted cells toward mesenchymal–epithelial transition (MET), as shown by fibronectin decreased expression (Fig. 2A,B) and E‐cadherin turnover at cell boundaries (Fig. 2B). Quantification of E‐cadherin levels at plasma membrane, as shown in diagram in Fig. 2C and Fig. S2B,C, confirmed decreased levels in UBE2T‐overexpressing cells, in contrast to the elevated plasma membrane E‐cadherin‐positive cells following UBE2T silencing.

We further confirmed EMT induction of UBE2T‐overexpressing cells by measuring cell‐free gap coverage over time (24 and 48 h). A higher gap closure was observed at 48 h in UBE2T‐overexpressing cells (*P* ≤ 0.0001 for both cell lines), whereas UBE2T silencing reverted to a decreased migration rate (*P* ≤ 0.0001 for both cell lines) (Fig. 2D and Fig. S2D). In addition, UBE2T overexpression enhanced the invasive potential of HCC cells (*P* ≤ 0.001 for HepG2 and *P* ≤ 0.0001 for Huh7), as confirmed by an 8‐fold increase in the number of invaded cells for both cell lines (Fig. 2E,F). Overall, our findings indicate the role of UBE2T in the maintenance of the epithelial integrity and subsequent EMT induction in HCC cells.

### 3.3 UBE2T triggers the activation of MAPK/ERK signaling pathway

**Fig. 3:**
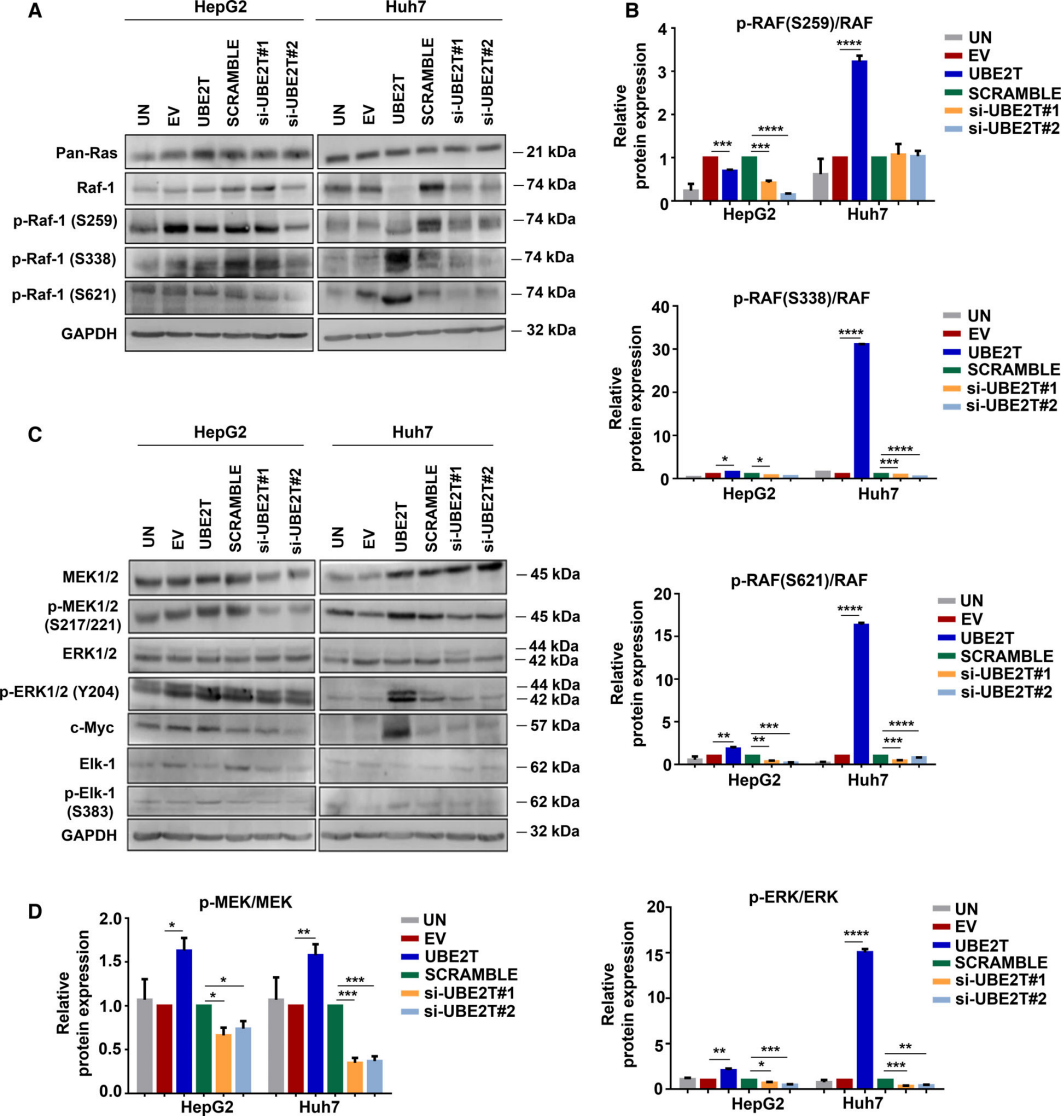
UBE2T enhances the MAPK/ERK signaling pathway. (A) Western blot analysis for the detection of protein levels of the upstream effectors of the MAPK/ERK pathway. (B) Quantification of the ratio of proteins: p‐RAF‐1 (S259)/RAF‐1, p‐RAF‐1(S338)/RAF‐1, p‐RAF‐1(S621)/RAF‐1 from (A). (C) Western blot analysis for the detection of protein levels of the downstream effectors of the MAPK/ERK pathway. (D) Quantification of the ratio of proteins: p‐MEK/MEK and p‐ERK/ERK from (C). Data shown are the mean ± SEM from *n* = 4 independent experiments, two‐tailed Student's *t*‐test (*P ≤ 0.05, **P ≤ 0.01, ***P ≤ 0.001, ****P ≤ 0.0001). Empty vector (EV)‐transfected cells were used as control for UBE2T ectopic overexpression (UBE2T), while nontargeting siRNA (SCRAMBLE) as control for UBE2T silencing (si‐UBE2T#1, si‐UBE2T#2). Untransfected cells (UN) were used as negative control. Normalization in western blot was performed to GAPDH.

The MAPK/ERK pathway cascade converts extracellular molecules such as growth factors, tumor‐promoting substances, and differentiation factors into intracellular signals regulating cell proliferation, differentiation, and survival [42]. Protein levels of Pan‐Ras (KRAS, HRAS and NRAS), the upstream effector of MAPK/ERK, and RAF (predominant form RAF‐1, linking kinase between RAS and MEK/ERK activation [43]), were assessed in UBE2T‐overexpressing and control cells (Fig. 3A,B and Fig. S3A). Next, we evaluated the levels of phosphorylated forms of RAF‐1 in three specific residues (S259, S338, and S621). S338 and S621 sites are required for the activation of RAF‐1 kinase, while S259 has been characterized as an inhibitory phosphorylation site [44]. Increased levels of p‐RAF‐1(S338)/RAF‐1 were observed in HepG2 (1.5‐fold, *P* ≤ 0.05) and extremely high levels in Huh7 UBE2T‐overexpressing cells (31‐fold, *P* ≤ 0.0001). A similar pattern of elevated expression of p‐RAF‐1(S621)/RAF‐1 was observed in HepG2 (1.8‐fold, *P* ≤ 0.01) and Huh7 UBE2T‐overexpressing cells (16‐fold, *P* ≤ 0.0001) (Fig. 3A,B).

Further confirmation of pathway activation after UBE2T overexpression was indicated by the increased levels of p‐MEK/MEK (1.6‐fold, *P* ≤ 0.05 for HepG2 and 1.6‐fold, *P* ≤ 0.01 for Huh7) and p‐ERK/ERK (2‐fold, *P* ≤ 0.01 for HepG2 and 15‐fold, *P* ≤ 0.0001 for Huh7). Interestingly, UBE2T silencing reverted the p‐MEK/MEK and p‐ERK/ERK levels in both cell lines (Fig. 3C,D). Selected downstream targets of MAPK/ERK signaling pathway, such as c‐Myc and p‐Elk‐1/Elk‐1, were also elevated after UBE2T overexpression (Fig. 3C and Fig. S3A). Overall, our results highlight the contribution of UBE2T in the regulation of MAPK/ERK signaling pathway.

### 3.4 UBE2T activates AKT/mTOR signaling pathway and upregulates β‐catenin protein expression

**Fig. 4:**
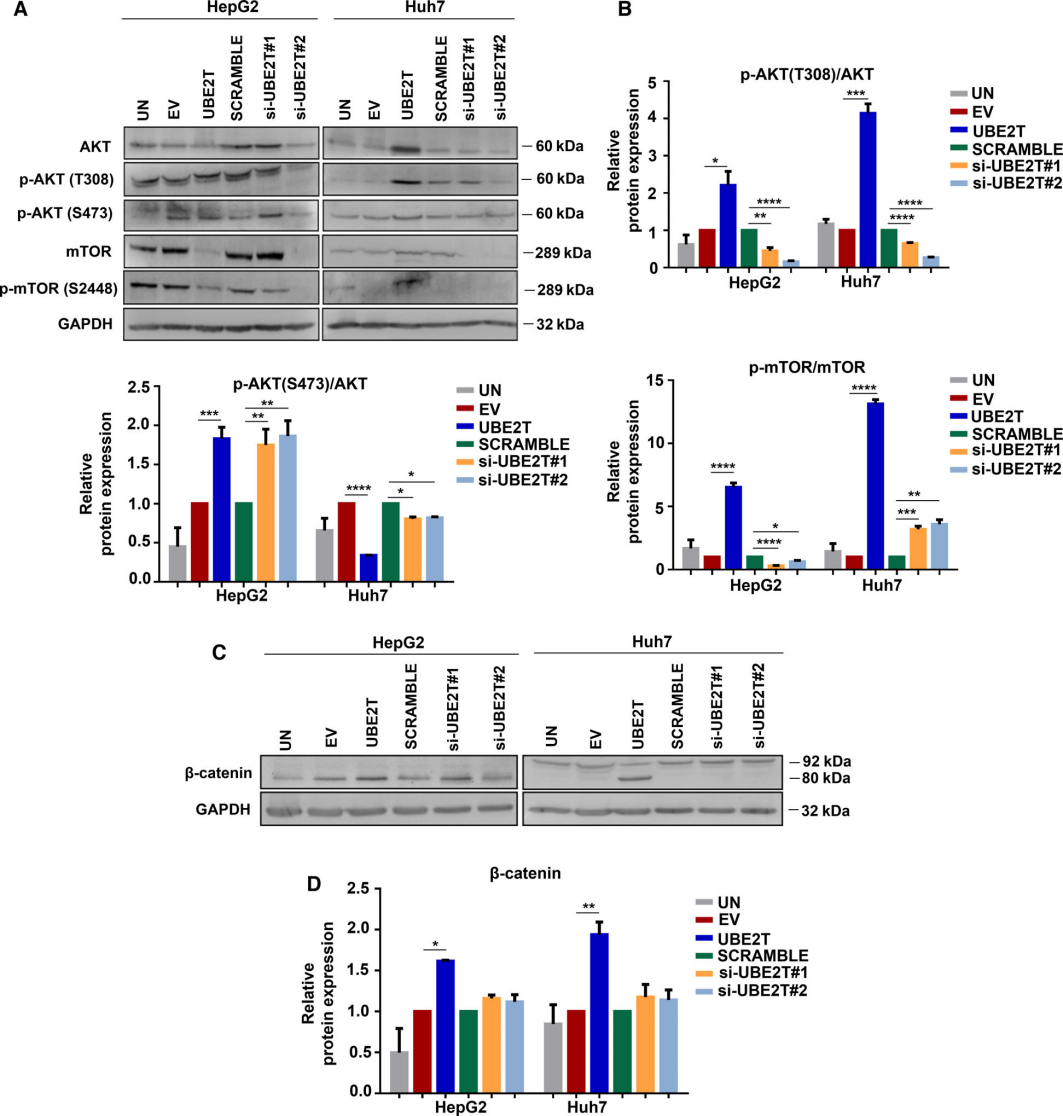
AKT/mTOR and β‐catenin activation by UBE2T overexpression. (A) Western blot analysis for the detection of protein levels of the components of the PI3K/AKT/mTOR pathway. (B) Quantification of the ratio of proteins: pAKT(T308)/AKT, pAKT(S473)/AKT, and pmTOR/mTOR from (A). (C) Western blot analysis for the detection of protein levels of total β‐catenin. (D) Quantification of β‐catenin from (C). Data shown are the mean ± SEM from *n* = 4 independent experiments, two‐tailed Student's *t*‐test (*P ≤ 0.05, **P ≤ 0.01, ***P ≤ 0.001, ****P ≤ 0.0001). Empty vector (EV)‐transfected cells were used as control for UBE2T ectopic overexpression (UBE2T), while non‐targeting siRNA (SCRAMBLE) as control for UBE2T silencing (si‐UBE2T#1, si‐UBE2T#2). Untransfected cells (UN) were used as negative control. Normalization in western blot was performed to GAPDH.

AKT/mTOR signaling pathway has been associated with many types of cancer, as all of its components are often deregulated [45]. AKT is a serine/threonine protein kinase that plays a crucial role in multiple cellular processes such as apoptosis, cell proliferation and migration [46]. T308 phosphorylation of AKT is essential, as it is located in the catalytic domain, while S473 phosphorylation triggers the full activation of the AKT protein kinase [9, 47, 48]. mTOR is a central regulator of mammalian metabolism and acts as a part of two structurally distinct complexes: mTORC1 and mTORC2 [49]. Notably, in both cell lines we observed increased ratio of pAKT(T308)/AKT following UBE2T overexpression, in contrast to decreased levels after UBE2T silencing. The expression pattern of pAKT(S473)/AKT was not the same in both cell lines, suggesting possible different mechanisms in the regulation of S473 phosphorylation of AKT. Τhe ratio of pmTOR/mTOR was extremely higher in UBE2T‐overexpressing cells, reaching a 6.5‐fold for HepG2 (*P* ≤ 0.0001) and a 13‐fold increase for Huh7 (*P* ≤ 0.0001), indicating elevated protein synthesis [50] (Fig. 4A,B).

We then explored whether UBE2T promoted Wnt/β‐catenin pathway activation. Wnt/β‐catenin pathway seems to be a promising target for molecular therapy, as new compounds are developing to inhibit this pathway [51, 52]. β‐Catenin is a multifunctional protein acting as an adhesion molecule in cooperation with E‐cadherin and as a transcription factor in tumor cell proliferation and metastasis [53, 54]. To clearly determine the role of β‐catenin in UBE2T‐driven HCC, we firstly measured its total protein levels, which were elevated after UBE2T overexpression. Interestingly, in Huh7 UBE2T‐overexpressing cells a cleaved fragment of β‐catenin, a band at 80 kDa was revealed (Fig. 4C,D). Overall, we remark the activation of the AKT/mTOR signaling pathway with an increase in β‐catenin protein levels in HCC cells following UBE2T overexpression.

### 3.5 UBE2T reinforces β‐catenin translocation into the nucleus leading to EMT cytoskeleton changes

**Fig. 5:**
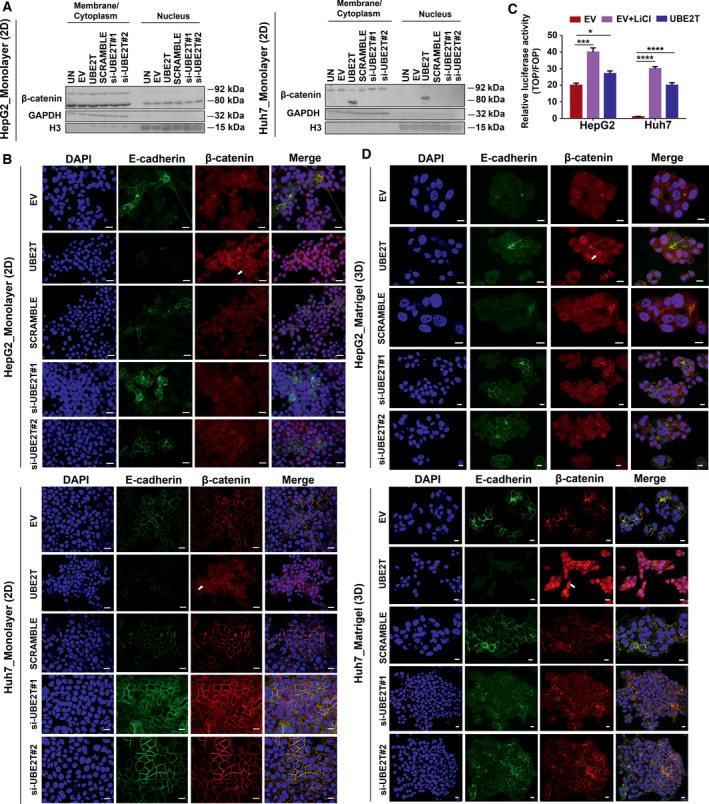
UBE2T accelerates β‐catenin translocation to the nucleus. (A) Western blot analysis of subcellular fractionated samples for the quantification of β‐catenin expression in membrane/cytoplasm or nucleus in monolayer (2D). (B) Single CLSM sections after immunostaining of UBE2T overexpression or silencing in HepG2/Huh7 (2D). Scale bar: 20 μm. E‐cadherin: green, β‐catenin: red, DAPI: blue. Arrows indicate nuclear β‐catenin. (C) TOPflash/FOPflash luciferase assay for the evaluation of β‐catenin transcriptional activation. LiCl was used as a Wnt/β‐catenin agonist. (D) Single CLSM sections after immunostaining of UBE2T overexpression or silencing in HepG2/Huh7‐embedded in Matrigel (3D). Scale bar: 10 μm. E‐cadherin: green, β‐catenin: red, DAPI: blue. Arrows indicate nuclear β‐catenin. Data shown are representative from *n* = 5 independent experiments, two‐tailed Student's *t*‐test (*P ≤ 0.05, ***P ≤ 0.001, ****P ≤ 0.0001). Empty vector (EV)‐transfected cells were used as control for UBE2T ectopic overexpression (UBE2T), while nontargeting siRNA (SCRAMBLE) as control for UBE2T silencing (si‐UBE2T#1, si‐UBE2T#2). Untransfected cells (UN) were used as negative control. Normalization was performed to GAPDH for membrane/cytoplasm and to histone H3 for nuclear fraction.

To explore the role of β‐catenin in HCC in accordance with UBE2T expression, we evaluated its subcellular localization. The increased presence of β‐catenin into the cytoplasm and nucleus concurrently is correlated with liver cancer progression and poor prognosis in contrast to plasma membrane β‐catenin, when it simultaneously binds to E‐cadherin acting as a cell–cell adhesion protein [55]. Cellular fractionation showed that UBE2T overexpression led to increased detection of β‐catenin in membrane/cytoplasm and nucleus fractions, in both HCC cell lines (Fig. 5A and Fig. S4A). In order to investigate the exact localization of β‐catenin and to further verify the results from immunoblotting, we performed confocal microscopy, initially in monolayer (2D) cells followed by analysis in Matrigel‐embedded cells (3D), as they recapitulate better the *in vivo* microenvironment [56].

In monolayer cultures, HepG2 UBE2T‐overexpressing cells showed a significant presence of β‐catenin in membrane/cytoplasm and nucleus (arrow in Fig. 5B), confirming the results of cellular fractionation experiments. HepG2 UBE2T‐silencing cells showed β‐catenin localization in the cytoplasm with strong E‐cadherin expression at cell boundaries (Fig. 5B). Interestingly, in Huh7 UBE2T‐overexpressing cells we observed a shift of β‐catenin from membrane to cytoplasm and its *de novo* expression in the nucleus (arrow in Fig. 5B). In Huh7 UBE2T‐silencing cells, a strong expression pattern of β‐catenin was observed in conjunction with a similar pattern of E‐cadherin expression at cell boundaries (Fig. 5B). Wnt/β‐catenin transcriptional activation was verified by performing the TOPflash/FOPflash luciferase assay. We observed that UBE2T overexpression cells showed increased luciferase activity, compared to control cells (EV) (Fig. 5C). Moreover, Huh7‐UBE2T cells reached the levels of LiCl (Wnt/β‐catenin agonist)‐treated cells, indicating the *de novo* nuclear appearance of β‐catenin.

In Matrigel‐embedded spheroid cultures, UBE2T overexpression in HepG2 cells led to an increased β‐catenin nuclear translocation (arrow in Fig. 5D) with a minimal amount localized at plasma membrane. Importantly, Huh7 UBE2T‐overexpressing cells presented an altered cellular morphology, from epithelial to mesenchymal, with elevated nuclear β‐catenin expression (arrow in Fig. 5D), in contrast to 2D culture conditions. Analysis of β‐catenin cellular distribution (%) performed in 2D and 3D showed a significant increase in a cell population with elevated nuclear β‐catenin signal in 3D, compared to 2D HCC UBE2T‐overexpressing cells (HepG2‐1.7‐fold, *P* ≤ 0.01 and Huh7‐16‐fold *P* ≤ 0.0001) (Fig. S4B,C). A more detailed observation showed an increase in a subpopulation of cells, positive for E‐cadherin, and nuclear β‐catenin compared with the total positive E‐cadherin population in 3D compared with 2D conditions (1.7‐fold, *P* ≤ 0.01 for HepG2 and 2.5‐fold, *P* ≤ 0.001 for Huh7 UBE2T‐overexpressing cells) (Fig. S4D), indicating that there is a proportion of cells, which may play a role during metastasis. Overall, all these data confirm the crucial involvement of UBE2T in β‐catenin subcellular localization, which is strongly correlated with cellular microenvironment. Cumulatively, our observations suggest that UBE2T leads to the activation of Wnt/β‐catenin pathway when conditions better mimic the 3D tumor microenvironment.

### 3.6 β‐catenin nuclear translocation is enhanced through an AKT‐independent mechanism

In order to investigate the molecular mechanisms of β‐catenin nuclear translocation, we generated stable monoclonal cell lines of UBE2T overexpression (C1, C2) in HepG2/Huh7, with their respective control cell lines (EV) (Fig. S5A). Firstly, we tested the involvement of Wnt ligands in β‐catenin activation using two chemical inhibitors, IWR‐1 and IWP‐2. IWR‐1, a tankyrase inhibitor of the Wnt/β‐catenin pathway, stabilizes Axin‐1 destruction complex resulting in β‐catenin phosphorylation/degradation [57, 58]. IWP‐2 inhibitor antagonizes the Wnt pathway, as it inactivates the membrane‐bound O‐acyltransferase porcupine (Porcn)‐mediated Wnt palmitoylation [59]. Regarding EMT induction and IWR‐1/IWP‐2 treatment total protein levels of β‐catenin, E‐cadherin and fibronectin were assessed (Fig. S5B,C).

**Fig. 6:**
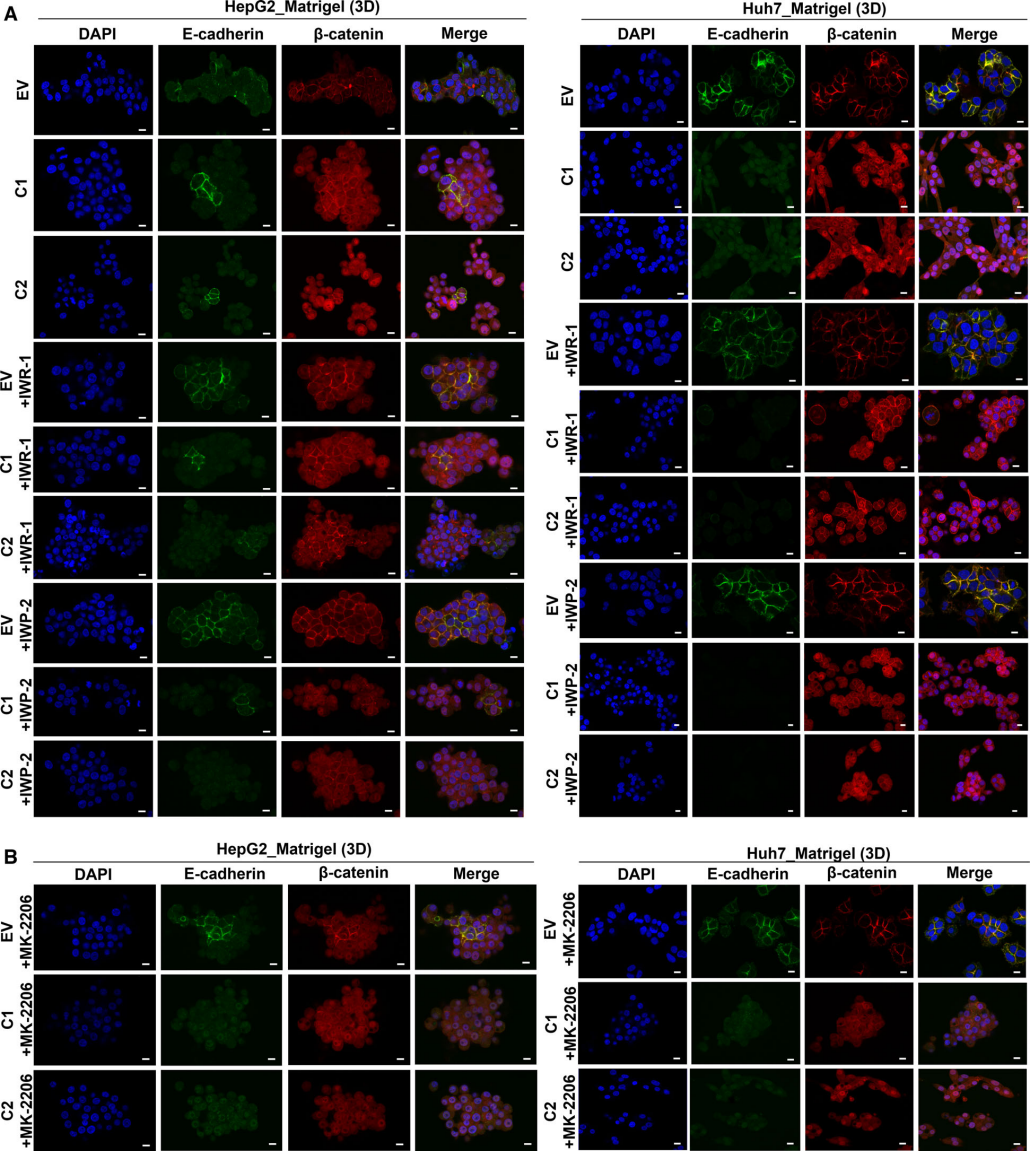
UBE2T enhances the activation of WNT/β‐catenin pathway through an AKT‐independent manner. (A) Single CLSM sections after immunostaining of UBE2T overexpression clones (C1, C2) and controls (EV)‐embedded in Matrigel (3D) with or without IWR‐1/IWP‐2 inhibitor treatment. Scale bar: 10 μm. E‐cadherin: green, β‐catenin: red, DAPI: blue. (B) Single CLSM sections after immunostaining of UBE2T overexpression clones (C1, C2) and controls (EV)‐embedded in Matrigel (3D) with MK‐2206 inhibitor treatment. Scale bar: 10 μm. E‐cadherin: green, β‐catenin: red, DAPI: blue. Data shown are representative from *n* = 5 independent experiments. Empty vector (EV) stable cell lines were used as control for UBE2T overexpression stable cell lines (C1, C2).

As for β‐catenin localization, subcellular fractionation showed that IWR‐1 treatment in HepG2 (C1, C2) led to elevated nuclear β‐catenin, while in Huh7 (C1, C2) resulted to a significant reduction of nuclear β‐catenin (*P* ≤ 0.0001), compared to untreated HepG2, Huh7 (C1, C2), respectively (Fig. S5D). As for IWP‐2 treatment, subcellular fractionation revealed reduced β‐catenin levels in the nuclei of HepG2 cells (C1, C2) (Fig. S5D), while Huh7 (C1, C2) showed no difference in the levels of β‐catenin in subcellular fractions (Fig. S5D). Intriguingly, both HCC (C1,C2) spheroids presented an overall reduction of nuclear β‐catenin following IWR‐1/IWP‐2 treatments (Fig. 6A), while Huh7 (C1, C2) showed simultaneously a complete cellular shape change (more epithelial‐like). Furthermore, the reduction of β‐catenin in UBE2T‐overexpressing cell lines seemed not to be adequate for E‐cadherin turnover at cell junctions, as its expression remained similar (Fig. 6A).

The role of AKT signaling pathway in β‐catenin regulation was evaluated utilizing the MK‐2206 inhibitor. MK‐2206 is an allosteric inhibitor for all three AKT isoforms and acts in a non‐ATP competitive manner [60]. MK‐2206 effectively downregulated the phosphorylation of AKT (T308, S473) in HepG2, while phosphorylation in Huh7 was inhibited only in S473 (Fig. S5E). Total levels of β‐catenin, E‐cadherin, and fibronectin were assessed (Fig. S5E). Subcellular fractionation showed that in both HCC (C1, C2)‐MK‐2206‐treated cell lines, nuclear β‐catenin was elevated, compared to EV (Fig. S5F). In 3D conditions, MK‐2206 led to elevated nuclear β‐catenin in HepG2, as opposed to Huh7 (C1, C2). E‐cadherin low levels were observed in HCC (C1, C2) (Fig. 6B), indicating that EMT remained enhanced in UBE2T‐driven HCC after MK‐2206 treatment.

Cumulatively, these data suggest that UBE2T is contributing toward the regulation of Wnt/β‐catenin pathway through an AKT‐independent manner, as AKT inhibition seems not to reverse UBE2T‐driven β‐catenin nuclear translocation. In addition, β‐catenin/E‐cadherin levels combined with alterations in cellular shape, being observed differently in 2D/3D conditions, support the necessity of different *in vitro* models for functional validation of each signaling pathway.

### 3.7 UBE2T‐mediated β‐catenin nuclear translocation and subsequent EMT is MAPK/ERK‐dependent

The role of the MAPK/ERK pathway in correlation with UBE2T expression was evaluated with SCH‐772984 inhibitor. SCH‐772984 is an ATP competitive ERK inhibitor, which effectively downregulates the MAPK/ERK signaling pathway in BRAF, MEK, and BRAF/MEK inhibitor‐resistant tumor models [61]. SCH‐772984 treatment led to a significant reduction of phosphorylated ERK levels in both cell lines. Furthermore, we noticed elevated E‐cadherin (the 135‐kDa proregion) and decreased fibronectin protein levels (Fig. S6A). Total β‐catenin protein levels in HepG2 remained unchanged, while an increase was observed in Huh7 after treatment (Fig. S6A). Subcellular fractionation showed reduced expression of β‐catenin in membrane/cytoplasm/nucleus in HepG2/Huh7 (C1, C2) and control cell lines (Fig. S6B). Decreased levels of β‐catenin in all fractions could be explained by abundant presence of β‐catenin into cell organelles (such as the endoplasmic reticulum, ER), which are difficult to disrupt during subcellular fractionation.

**Fig. 7:**
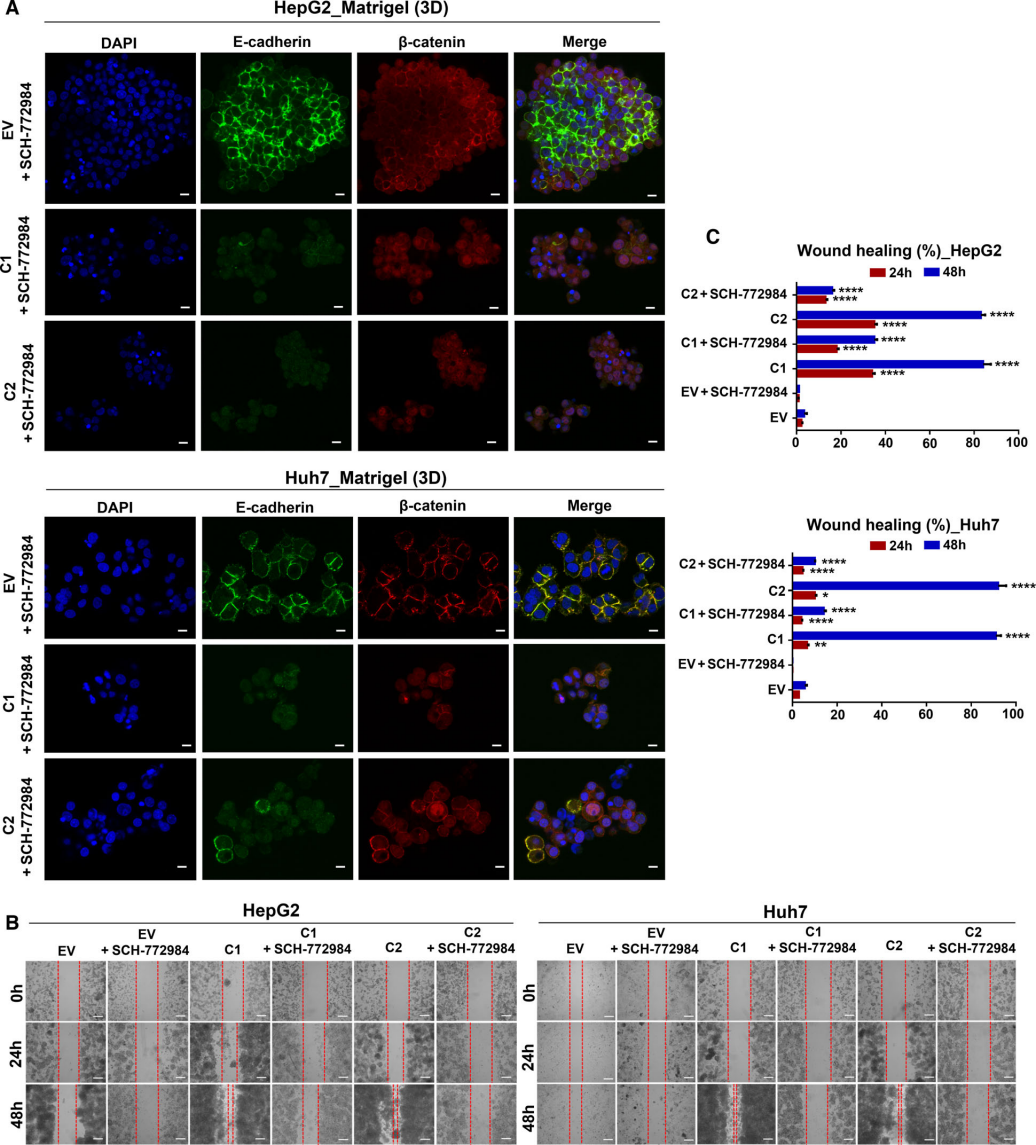
MAPK/ERK signaling pathway plays a central role in UBE2T‐driven EMT. (A) Single CLSM sections after immunostaining of UBE2T overexpression clones (C1, C2) and controls (EV)‐embedded in Matrigel (3D) with SCH‐772984 inhibitor treatment. Scale bar: 10 μm. E‐cadherin: green, β‐catenin: red, DAPI: blue. (B) Scratch assay captured at 24 and 48 h of EV and UBE2T overexpression clones (C1, C2) with or without SCH‐772984 inhibitor treatment. Scale bar: 200 μm. (C) Quantification of gap closure at 24 and 48 h from (B). Data shown are representative from *n* = 5 independent experiments, two‐tailed Student's *t*‐test (*P ≤ 0.05, **P ≤ 0.01, ****P ≤ 0.0001). Empty vector (EV) stable cell lines were used as control for UBE2T overexpression stable cell lines (C1, C2).

SCH‐772984 treatment in 3D culture conditions resulted in an overall reduction of β‐catenin (cytoplasmic and nuclear; Fig. 7A). A significant observation was that in 3D conditions, E‐cadherin levels were elevated at plasma membrane after SCH‐772984 treatment only in EV cell lines, in contrast to its decreased levels in UBE2T clones.

E‐cadherin trafficking at plasma membrane is frequently related to multiple post‐translational modifications, which can inhibit its export from the ER [62, 63], a notion that is supported by our previous findings regarding a possible β‐catenin presence in subcellular fractions after SCH‐772984 treatment. It is known though that E‐cadherin/β‐catenin complexes are formed cotranslationally in ER/Golgi compartments, prior to cleavage of E‐cadherin proregion [64]. However, we suggest that although E‐cadherin levels were similar in UBE2T clones, EMT was suppressed indicating by decreased nuclear β‐catenin in SCH‐772984‐treated cell lines.

Further confirmation that UBE2T‐mediated β‐catenin translocation and subsequent EMT is MAPK/ERK‐dependent was provided when we assessed the migratory capability in HepG2/Huh7 (C1, C2), compared to control cells with or without SCH‐772984 inhibitor. Migratory ability was significantly increased for HepG2 (C1, C2, *P* ≤ 0.0001) and for Huh7 (C1, C2, *P* ≤ 0.0001) at 48 h, while SCH‐772984 treatment led to its reversion (Fig. 7B,C) confirming the notion that UBE2T‐mediated EMT is MAPK/ERK‐dependent.

## 4 Discussion

Although liver cancer is one of the most malignant tumors worldwide, the molecular mechanisms underlying its initiation and progression are not well‐understood [3, 4, 5]. UBE2T has been characterized as an oncogene in many types of cancer, including HCC [29, 30, 31, 32, 33]. However, the involvement of UBE2T in EMT induction in HCC has not been investigated. In our study, we have shown that UBE2T in HCC cells in 2D and 3D culture conditions regulated β‐catenin nuclear translocation and led to subsequent induction of EMT through a MAPK/ERK‐dependent activation.

In addition, UBE2T regulated proliferation and cell cycle progression, as its overexpression led HepG2 to a G2/M arrest and polyploidy, while Huh7 to an accelerated S‐phase progression with a rapid bypassing of G2/M phase and subsequent hypodiploidy. This is a common observed phenomenon, when increased expression of oncogenes such as c‐Myc or RAS drive cancer cells through aberrant mitosis to both death‐in‐mitosis, postmitotic apoptosis, and chromosomal instability [65, 66, 67]. In conjunction, UBE2T was reported to promote CHK1 activation and G2/M arrest after ionizing radiation via H2AX monoubiquitination in HCC cells [68].

In accordance with other reports, UBE2T has been connected to EMT in several types of cancers, such as glioblastoma, gastric, and lung cancer [37, 38, 39]. In our study, we further demonstrated that UBE2T‐mediated EMT induction was correlated with increased nuclear translocation of β‐catenin in HepG2 and *de novo* elevated nuclear expression in Huh7 cells, leading to its transcriptional activation. We showed that nuclear translocation of β‐catenin was affected by the cellular microenvironment, as in 3D spheroids which better recapitulate the *in vivo* state, its expression was clearly elevated in UBE2T‐overexpressing cells. More specifically, UBE2T led to increased Wnt/β‐catenin activation, independently of β‐catenin mutational status in HCC cells.

IWR‐1/IWP‐2 inhibitor treatments in both cell lines, when cultured in 3D conditions, showed gradual decrease in UBE2T‐driven nuclear β‐catenin translocation. The strong β‐catenin membrane relocalization after IWR‐1 treatment following UBE2T overexpression or not implies that β‐catenin nuclear translocation and subcellular localization are possibly regulated synergistically by Wnt and other signaling pathways. Aberrant Wnt signaling which is triggered by UBE2T overexpression is a feature of advanced HCC characterized by the activation of other main oncogenic pathways such as AKT/mTOR, MAPK/ERK, and TGF‐β, which are affected differently in 2D and 3D conditions in many cancers including HCC [69, 70, 71]. As a consequence, IWR‐1 blocked effectively the nuclear translocation of β‐catenin and the acquisition of an augmented oncogenic phenotype, as previous studies have revealed, independently of UBE2T [72, 73, 74, 75]. However, in many cases the presence of only β‐catenin at the plasma membrane without E‐cadherin re‐expression has been related with advanced stages of HCC, as plasma membrane E‐cadherin recruits β‐catenin away from Wnt pathway activation [12, 76, 77].

Many studies have shown that UBE2T affects AKT/GSK3‐β, as well as Wnt/β‐catenin pathway [39, 78] in many types of cancer including HCC [29, 32]. However, no correlation has ever been made so far between UBE2T expression in HCC and MAPK/ERK pathway. MAPK/ERK pathway is often deregulated in HCC [79], while Zhang *et al*. [80] remarked the MAPK/ERK regulation in non‐small‐lung cancer by another E2 enzyme, UBE2C. Our work, collectively suggests that UBE2T enhanced MAPK/ERK activation, while MAPK/ERK inhibition reduced nuclear β‐catenin expression and subsequent EMT in UBE2T‐overexpressing HCC cells, a notion also supported by other studies in the regulation of β‐catenin degradation, localization, and cancer progression [81, 82, 83].

## 5 Conclusions

In summary, the present work showed that UBE2T overexpression induced EMT in HCC cell lines and activated EMT‐associated signaling pathways: MAPK/ERK, AKT/mTOR, and Wnt/β‐catenin. More specifically, we found for the first time that UBE2T enhanced β‐catenin nuclear translocation and subsequent EMT mainly through a MAPK/ERK‐dependent mechanism. We should not, however, exclude the possibility of non‐enzymatic functions of UBE2T (e.g., as scaffolding partner). Further studies which will investigate the contribution of UBE2T and its protein partners to E‐cadherin/β‐catenin post‐translational process and recruitment to plasma membrane in correlation with the regulation of MAPK/ERK could be a promising strategy for unraveling newly molecular therapies against HCC.

## Conflict of interest

The authors declare no conflict of interest.

### Peer Review

The peer review history for this article is available at https://publons.com/publon/10.1002/1878‐0261.13111.

## Author contributions

EL designed, performed the experiments, analyzed the data, and wrote the manuscript. PM performed bioinformatics analysis. EP contributed to confocal microscopy experiments. DD designed and supervised the study. All authors read and approved the final manuscript.

## Supporting information


**Fig. S1.** Quantification of growth promoting traits of HCC cells in accordance with UBE2T expression. A. Western blot analysis for the quantification of UBE2T expression in HepG2/Huh7. B. Quantification of UBE2T expression from (A). C. Representative images of colony formation assay. Scale bar: 200 μm. D. Representative images of calcein‐AM and EthD‐1 staining. Scale bar: 20 μm. Calcein‐AM: green, EthD‐1: red. E. Table with the cell percentage (%) at cell cycle phases. F. Apoptosis quantification with PI/Annexin V staining. Etoposide was used as an apoptosis inducer. Data shown are the mean ± SEM from n = 3 independent experiments, two‐tailed Student's t test (** p ≤ 0.01, **** p ≤ 0.0001). Empty vector (EV) transfected cells were used as control for UBE2T ectopic overexpression (UBE2T), while non‐targeting siRNA (SCRAMBLE) as control for UBE2T silencing (si‐UBE2T#1, si‐UBE2T#2). Untransfected cells (UN) were used as negative control. Normalization in western blot was performed to GAPDH.
**Fig. S2.** Quantification of EMT in HCC cells in accordance with UBE2T expression. A. Quantification of E‐cadherin, Fibronectin and Slug expression in HepG2/Huh7. B. Western blot analysis of subcellular fractionated samples for the quantification of E‐cadherin expression in cytoplasm and plasma membrane in monolayer (2D). C. Quantification of E‐cadherin levels from (B). D. Quantification of wound healing (%). Data shown are the mean ± SEM from n = 5 independent experiments, two‐tailed Student's t test (* p ≤ 0.05, ** p ≤ 0.01, *** p ≤ 0.001, **** p ≤ 0.0001). Empty vector (EV) transfected cells were used as control for UBE2T ectopic overexpression (UBE2T), while non‐targeting siRNA (SCRAMBLE) as control for UBE2T silencing (si‐UBE2T#1, si‐UBE2T#2). Untransfected cells (UN) were used as negative control. Normalization in western blot of whole‐cell lysates was performed to GAPDH. Normalization in western blot of subcellular fractionated samples was performed to Ponceau staining and α‐tubulin was used as cytoplasmic marker, whereas Histone H3 as nuclear.
**Fig. S3.** Quantification of expression of MAPK/ERK components in accordance with UBE2T expression. A. Quantification of Pan‐Ras, c‐Myc and p‐Elk‐1/Elk‐1. Data shown are the mean ± SEM from n = 4 independent experiments, two‐tailed Student's t test (* p ≤ 0.05, ** p ≤ 0.01, *** p ≤ 0.001, **** p ≤ 0.0001). Empty vector (EV) transfected cells were used as control for UBE2T ectopic overexpression (UBE2T), while non‐targeting siRNA (SCRAMBLE) as control for UBE2T silencing (si‐UBE2T#1, si‐UBE2T#2). Untransfected cells (UN) were used as negative control. Normalization in western blot was performed to GAPDH.
**Fig. S4.** Quantification of β‐catenin protein levels at different cellular compartments. A. Quantification of β‐catenin expression in membrane/cytoplasm or nucleus at subcellular fractionated samples from Western blot analysis. Normalization was performed to GAPDH for membrane/cytoplasmic and to Histone H3 for nuclear fraction. B. Quantification of the cellular distribution of β‐catenin at single CLSM sections after immunostaining of UBE2T overexpression or silencing in HepG2/Huh7 monolayer (2D). C. Quantification of the cellular distribution of β‐catenin at single CLSM sections after immunostaining of UBE2T overexpression or silencing in HepG2/Huh7‐emdedded in matrigel (3D). D. Quantification of the percentage of the subpopulation of cells which is E‐cadherin(+) and nuclear β‐catenin(+) to total E‐cadherin(+) cells in 2D and 3D. Data shown are the mean ± SEM from n = 5 independent experiments, two‐tailed Student's t test (* p ≤ 0.05, ** p ≤ 0.01, *** p ≤ 0.001, **** p ≤ 0.0001). Empty vector (EV) transfected cells were used as control for UBE2T ectopic overexpression (UBE2T), while non‐targeting siRNA (SCRAMBLE) as control for UBE2T silencing (si‐UBE2T#1, si‐UBE2T#2). Untransfected cells (UN) were used as negative control. N:nucleus, MC:membrane/cytoplasm, NMC:nucleus/membrane/cytoplasm.
**Fig. S5.** The effect of IWR‐1, IWP‐2 and MK‐2206 treatments on EMT process after UBE2T overexpression. Α. Western blots and bar graph showing the expression levels of UBE2T in UBE2T‐overexpression stable cell lines (C1, C2), compared to parental (EV). B. Western blots and bar graphs showing the expression levels of β‐catenin, E‐cadherin and Fibronectin after ΙWR‐1 treatment. C. Western blots and bar graphs showing the expression levels of β‐catenin, E‐cadherin and Fibronectin after ΙWP‐2 treatment. D. Western blot analysis and quantification graphs of subcellular fractionated samples for the quantification of β‐catenin expression in membrane/cytoplasm or nucleus after IWR‐1 and IWP‐2 treatment. E. Western blots and bar graphs showing the expression levels of AKT, pAKT (T308, S473), E‐cadherin, β‐catenin and Fibronectin after MK‐2206 treatment. F. Western blot analysis and quantification graphs of subcellular fractionated samples for the quantification of β‐catenin expression in membrane/cytoplasm or nucleus after MK‐2206 treatment. Data shown are the mean ± SEM from n = 5 independent experiments, two‐tailed Student's t test (* p ≤ 0.05, ** p ≤ 0.01, *** p ≤ 0.001, **** p ≤ 0.0001). Empty vector (EV) stable cell lines were used as control for UBE2T‐overexpression stable cell lines (C1, C2). Normalization in western blot of whole‐cell lysates was performed to GAPDH. Normalization was performed to GAPDH for membrane/cytoplasmic and to Histone H3 for nuclear fraction. N:nucleus, MC:membrane/cytoplasm.
**Fig. S6.** The effect of SCH‐772984 treatment on EMT process after UBE2T overexpression. Α. Western blots and bar graphs showing the expression levels of p‐ERK/ERK, E‐cadherin, β‐catenin and Fibronectin after SCH‐772984 treatment. Β. Western blot analysis and quantification graphs of subcellular fractionated samples for the quantification of β‐catenin expression in membrane/cytoplasm or nucleus after SCH‐772984 treatment. Data shown are the mean ± SEM from n = 5 independent experiments, two‐tailed Student's t test (* p ≤ 0.05, ** p ≤ 0.01, *** p ≤ 0.001, **** p ≤ 0.0001). Empty vector (EV) stable cell lines were used as control for UBE2T overexpression stable cell lines (C1, C2). Normalization in western blot of whole‐cell lysates was performed to GAPDH. Normalization was performed to GAPDH for membrane/cytoplasmic and to Histone H3 for nuclear fraction. N:nucleus, MC:membrane/cytoplasm.
**Table S1.** List of primary and secondary antibodies used in this study.

## Data Availability

The data that support the findings of this study are available in the figures and the supplementary material of this article.
