# Supplementary material for: UBE2T promotes β‐catenin nuclear translocation in hepatocellular carcinoma through MAPK/ERK‐dependent activation
Source: Mol Oncol. 2022 Mar 16;16(8):1694–713. doi: 10.1002/1878-0261.13111 (PMC9019890; doi:10.1002/1878-0261.13111)
Supplement: Supplementary file 1 — Fig. S1. Quantification of growth promoting traits of HCC cells in accordance with UBE2T expression. A. Western blot analysis for the quantification of UBE2T expression in HepG2/Huh7. B. Quantification of UBE2T expression from (A). C. Representative images of colony formation assay. Scale bar: 200 μm. D. Representative images of calcein‐AM and EthD‐1 staining. Scale bar: 20 μm. Calcein‐AM: green, EthD‐1: red. E. Table with the cell percentage (%) at cell cycle phases. F. Apoptosis quantification with PI/Annexin V staining. Etoposide was used as an apoptosis inducer. Data shown are the mean ± SEM from n = 3 independent experiments, two‐tailed Student's t test (** p ≤ 0.01, **** p ≤ 0.0001). Empty vector (EV) transfected cells were used as control for UBE2T ectopic overexpression (UBE2T), while non‐targeting siRNA (SCRAMBLE) as control for UBE2T silencing (si‐UBE2T#1, si‐UBE2T#2). Untransfected cells (UN) were used as negative control. Normalization in western blot was performed to GAPDH. Fig. S2. Quantification of EMT in HCC cells in accordance with UBE2T expression. A. Quantification of E‐cadherin, Fibronectin and Slug expression in HepG2/Huh7. B. Western blot analysis of subcellular fractionated samples for the quantification of E‐cadherin expression in cytoplasm and plasma membrane in monolayer (2D). C. Quantification of E‐cadherin levels from (B). D. Quantification of wound healing (%). Data shown are the mean ± SEM from n = 5 independent experiments, two‐tailed Student's t test (* p ≤ 0.05, ** p ≤ 0.01, *** p ≤ 0.001, **** p ≤ 0.0001). Empty vector (EV) transfected cells were used as control for UBE2T ectopic overexpression (UBE2T), while non‐targeting siRNA (SCRAMBLE) as control for UBE2T silencing (si‐UBE2T#1, si‐UBE2T#2). Untransfected cells (UN) were used as negative control. Normalization in western blot of whole‐cell lysates was performed to GAPDH. Normalization in western blot of subcellular fractionated samples was performed to Ponceau staining and α‐tub [file MOL2-16-1694-s001.pdf]

**Table S1.** List of primary and secondary antibodies used in this study.

| Antibodies | Antigen | Clone | Species | Company | Cat. number | Dilution |
|---|---|---|---|---|---|---|
| **Primary antibodies** | UBE2T | 4E5 | mAb mouse | Santa Cruz Biotechnology | sc-100623 | 1:200 |
| | E-cadherin | H-108 | pAb rabbit | Santa Cruz Biotechnology | sc-7870 | 1:500 |
| | β-catenin | E5 | mAb mouse | Santa Cruz Biotechnology | sc-7963 | 1:200 |
| | Fibronectin | EP5 | mAb mouse | Santa Cruz Biotechnology | sc-8422 | 1:500 |
| | Slug | A7 | mAb mouse | Santa Cruz Biotechnology | sc-166476 | 1:200 |
| | α-tubulin | B7 | mAb mouse | Santa Cruz Biotechnology | sc-5286 | 1:500 |
| | AKT | C67E7 | mAb rabbit | Cell Signaling | 4691 | 1:1000 |
| | p-AKT (T308) | D25E6 | mAb rabbit | Cell Signaling | 13038 | 1:500 |
| | p-AKT (S473) | D9E | mAb rabbit | Cell Signaling | 4060 | 1:500 |
| | mTOR | 7C10 | mAb rabbit | Cell Signaling | 2983 | 1:1000 |
| | pmTOR (S2448) | | pAb rabbit | Cell Signaling | 2971 | 1:500 |
| | c-MYC | 9E10 | mAb mouse | Santa Cruz Biotechnology | sc-40 | 1:200 |
| | Pan Ras | C-4 | mAb mouse | Santa Cruz Biotechnology | sc-166691 | 1:200 |
| | Raf-1 | C-12 | pAb rabbit | Santa Cruz Biotechnology | sc-133 | 1:200 |
| | p-Raf-1/c-RAF (S259) | | pAb rabbit | Cell Signaling | 9421 | 1:500 |
| | p-Raf-1/c-RAF (S338) | 56A6 | mAb rabbit | Cell Signaling | 9427 | 1:500 |
| | p-Raf-1 (S621) | E-1 | mAb mouse | Santa Cruz Biotechnology | sc-271929 | 1:500 |

**Table S1.** List of primary and secondary antibodies used in this study.

| Antibodies | Antigen | Clone | Species | Company | Cat. number | Dilution |
|---|---|---|---|---|---|---|
| **Primary antibodies** | MEK1/2 | | pAb rabbit | Cell Signaling | 9122 | 1:1000 |
| | p-MEK1/2 (S217/221) | 41G9 | mAb rabbit | Cell Signaling | 9154 | 1:1000 |
| | ERK1/2 | C-9 | mAb mouse | Santa Cruz Biotechnology | sc-514302 | 1:500 |
| | p-ERK1/2 (Y204) | E-4 | mAb mouse | Santa Cruz Biotechnology | sc-7383 | 1:500 |
| | Bcl-2 | C-2 | mAb mouse | Santa Cruz biotechnology | sc-7382 | 1:100 |
| | Bax | B-9 | mAb mouse | Santa Cruz Biotechnology | sc-7480 | 1:200 |
| | Elk-1 | E-5 | mAb mouse | Santa Cruz Biotechnology | sc-365876 | 1:100 |
| | p-Elk-1 | B-4 | mAb mouse | Santa Cruz Biotechnology | sc-8406 | 1:100 |
| | GAPDH | 0411 | mAb mouse | Santa Cruz Biotechnology | sc-47724 | 1:2000 |
| | Histone H3 | 1G1 | mAb mouse | Santa Cruz Biotechnology | sc-517576 | 1:10000 |
| **Secondary antibodies** | anti-mouse HRP | | | Santa Cruz Biotechnology | sc-516102 | 1:3000 |
| | anti-rabbit HRP | | mouse | Santa Cruz Biotechnology | sc-2357 | 1:3000 |
| | anti-rabbit Alexa Fluor 488 | | pAb goat | Invitrogen | A11008 | 1:500 |
| | anti-mouse Alexa Fluor 555 | | pAb donkey | Invitrogen | A31570 | 1:500 |

# Figure S1.

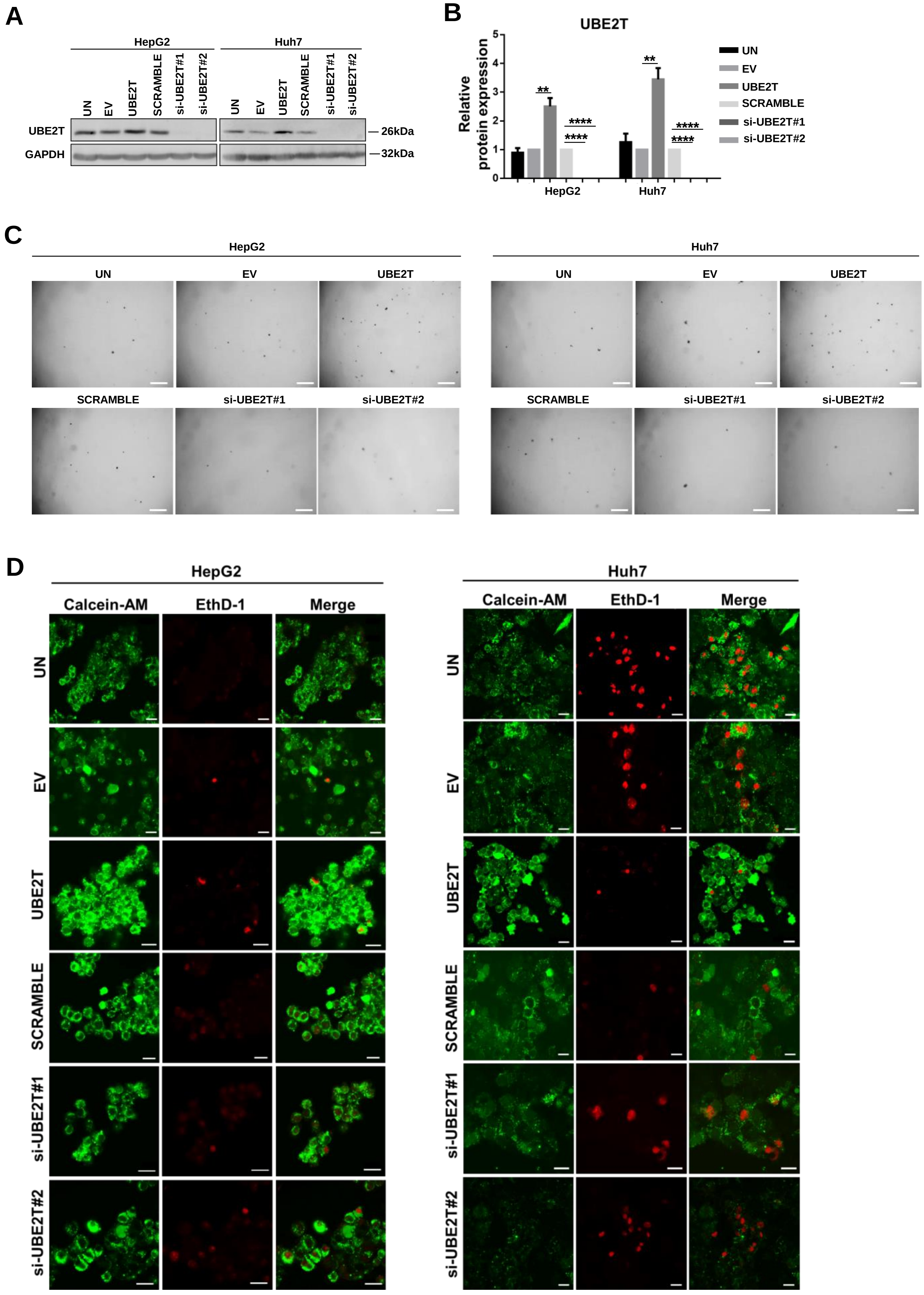
A
HepG2
Huh7
UN
EV
UBE2T
SCRAMBLE
si-UBE2T#1
si-UBE2T#2
UBE2T
GAPDH
26kDa
32kDa
B
UBE2T
Relative protein expression
HepG2
Huh7
UN
EV
UBE2T
SCRAMBLE
si-UBE2T#1
si-UBE2T#2
C
HepG2
Huh7
UN
EV
UBE2T
SCRAMBLE
si-UBE2T#1
si-UBE2T#2
D
HepG2
Huh7
Calcein-AM
EthD-1
Merge
UN
EV
UBE2T
SCRAMBLE
si-UBE2T#1
si-UBE2T#2

# Figure S1.

## E

| Treatment Group (HepG2)/Cell cycle phases | SubG1 | G0/G1 | S | G2/M | Polyploid |
|---|---|---|---|---|---|
| UN | 22.2±4.4 | 52.6±0.9 | 8.9±1.0 | 14.6±4.9 | 2.2±0.9 |
| EV | 22.4±0.2 | 52.0±0.2 | 8.7±0.6 | 14.7±1.0 | 1.9±0.6 |
| UBE2T | 9.9±3.8 | 44.0±4.2 | 3.5±1.1 | 27.7±3.5 | 16.3±4.0 |
| SCRAMBLE | 18.8±4.5 | 56.0±0.4 | 12.1±1.2 | 12.2±4.4 | 1.8±0.3 |
| si-UBE2T#1 | 21.9±3.2 | 57.7±1.4 | 9.9±1.7 | 11.5±0.9 | 1.3±0.7 |
| si-UBE2T#2 | 14.4±2.7 | 61.1±2.4 | 7.7±1.6 | 15.1±4.0 | 1.5±0.8 |
| **Treatment Group (Huh7)/Cell cycle phases** | **SubG1** | **G0/G1** | **S** | **G2/M** | **Polyploid** |
| UN | 8.1±1.8 | 47.5+3.2 | 14.6±2.3 | 19.5±3.1 | 10.3±2.4 |
| EV | 16.0±2.5 | 38.3±7.2 | 11.2±1.6 | 23.9±4.8 | 10.0±3.1 |
| UBE2T | 48.4±6.2 | 17.6±5.6 | 11.1±2.5 | 16.3±4.5 | 6.2±3.4 |
| SCRAMBLE | 15.9±3.9 | 42.7±4.1 | 9.1±1.4 | 20.4±5.6 | 11.2±3.4 |
| si-UBE2T#1 | 16.1±2.3 | 55.5±2.5 | 7.6±0.9 | 12.6±5.5 | 6.8±3.1 |
| si-UBE2T#2 | 16.8±3.8 | 59.5±2.6 | 5.8±0.9 | 12.8±4.8 | 4.6±2.5 |

## F

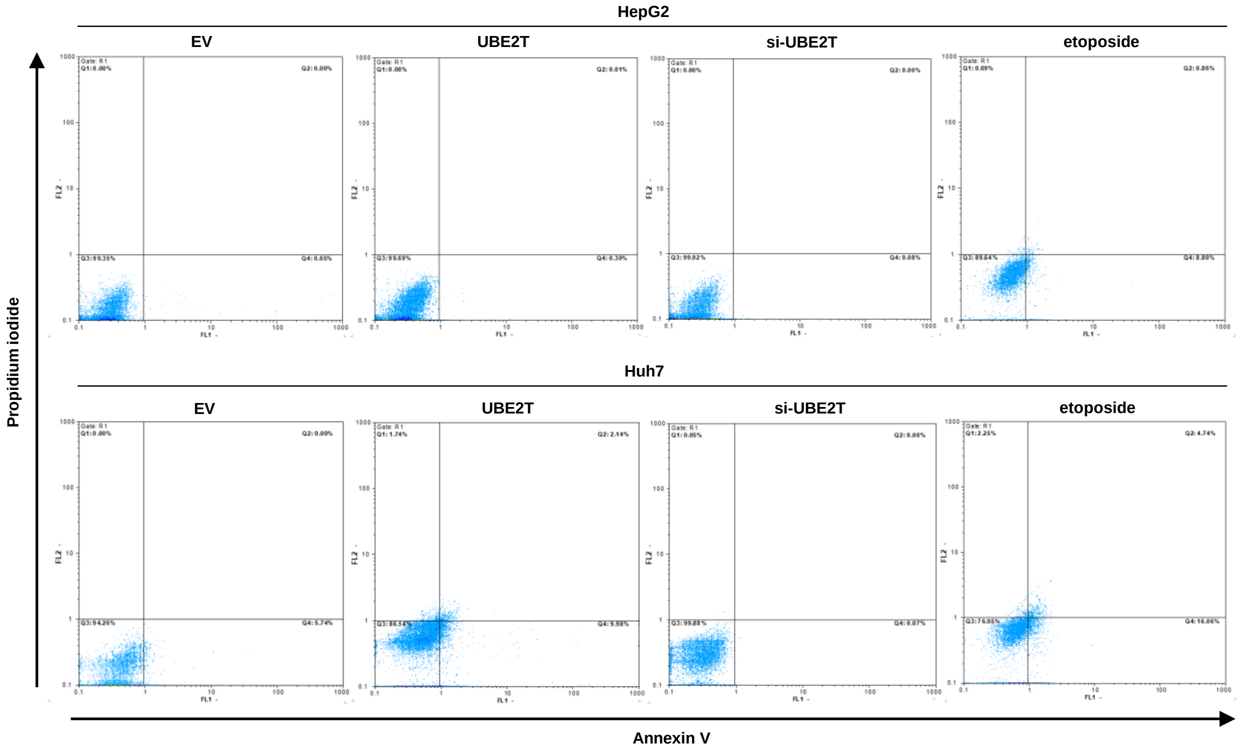


| Treatment Group (HepG2) | Live (Pi$^-$/Annexin V$^-$) | Early apoptotic (Pi$^-$/Annexin V$^+$) | Late apoptotic (Pi$^+$/Annexin V$^+$) | Necrotic (Pi$^+$/Annexin V$^-$) |
|---|---|---|---|---|
| EV | 99±0.5 | 1±0.05 | 0±0 | 0±0 |
| UBE2T | 99.5±0.1 | 0.5±0.02 | 0.1±0.002 | 0±0 |
| si-UBE2T | 99.8±0.1 | 0.05±0.01 | 0±0 | 0±0 |
| etoposide | 89±0.3 | 9±0.2 | 1±0.2 | 1±0.5 |
| **Treatment Group (Huh7)** | | | | |
| EV | 94±0.9 | 6±0.3 | 0±0 | 0±0 |
| UBE2T | 85±1.1 | 10±0.2 | 2±0.2 | 2±0.3 |
| si-UBE2T | 99.9±0.2 | 0.1±0.01 | 0±0 | 0.1±0.05 |
| etoposide | 75±2 | 17±1 | 5±0.5 | 2.5±0.4 |

**Figure S2.**

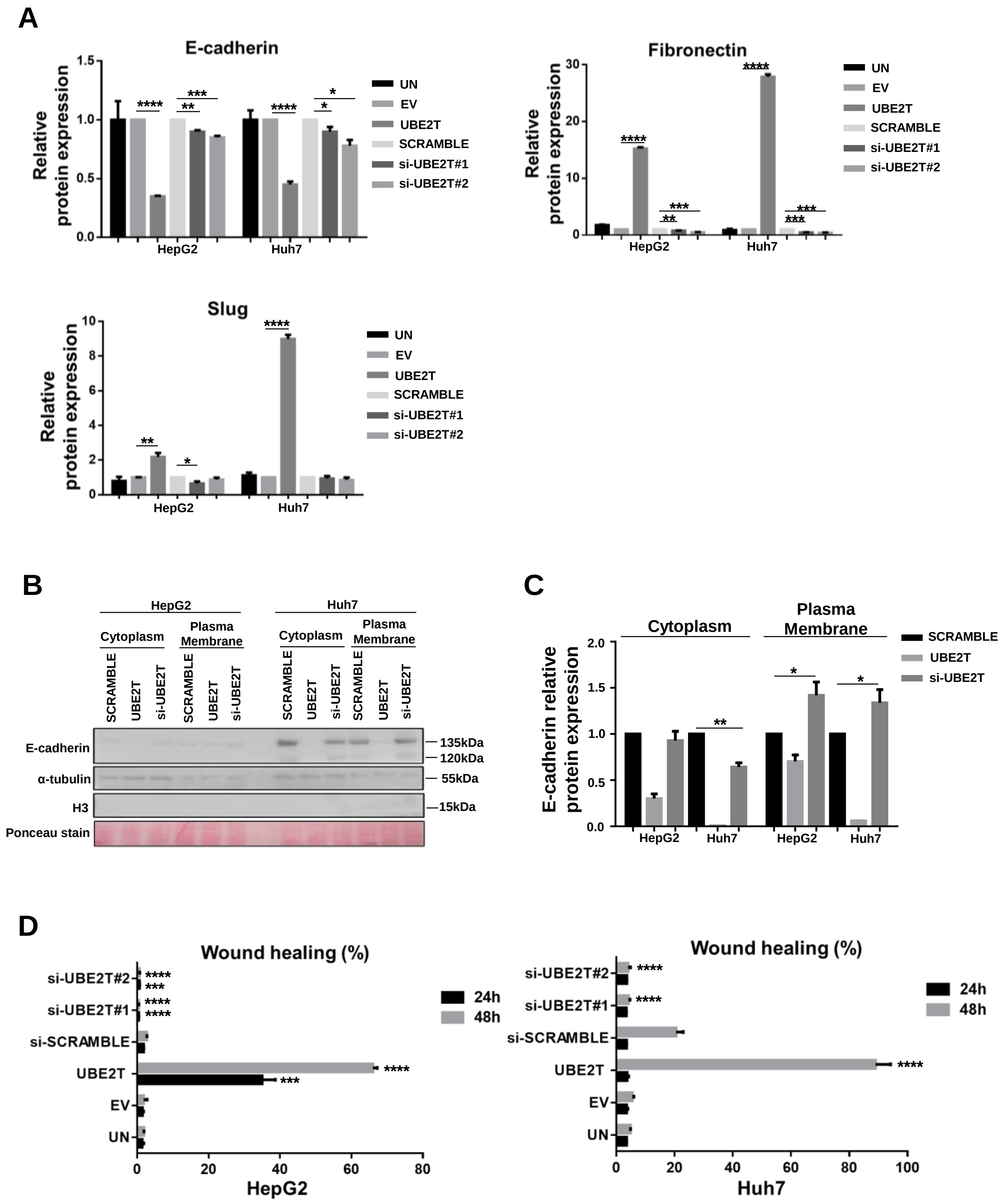
A
E-cadherin
Relative protein expression
1.5
1.0
0.5
0.0
****
***
**
****
*
*
HepG2
Huh7
UN
EV
UBE2T
SCRAMBLE
si-UBE2T#1
si-UBE2T#2
Fibronectin
30
20
10
0
****
****
***
**
***
***
Slug
10
8
6
4
2
0
****
**
*
B
HepG2
Huh7
Cytoplasm
Plasma Membrane
SCRAMBLE
UBE2T
si-UBE2T
E-cadherin
α-tubulin
H3
Ponceau stain
135kDa
120kDa
55kDa
15kDa
C
Cytoplasm
Plasma Membrane
E-cadherin relative protein expression
2.0
1.5
1.0
0.5
0.0
SCRAMBLE
UBE2T
si-UBE2T
D
Wound healing (%)
si-UBE2T#2
si-UBE2T#1
si-SCRAMBLE
UBE2T
EV
UN
24h
48h
0
20
40
60
80
100
HepG2
Huh7

## Figure S3.

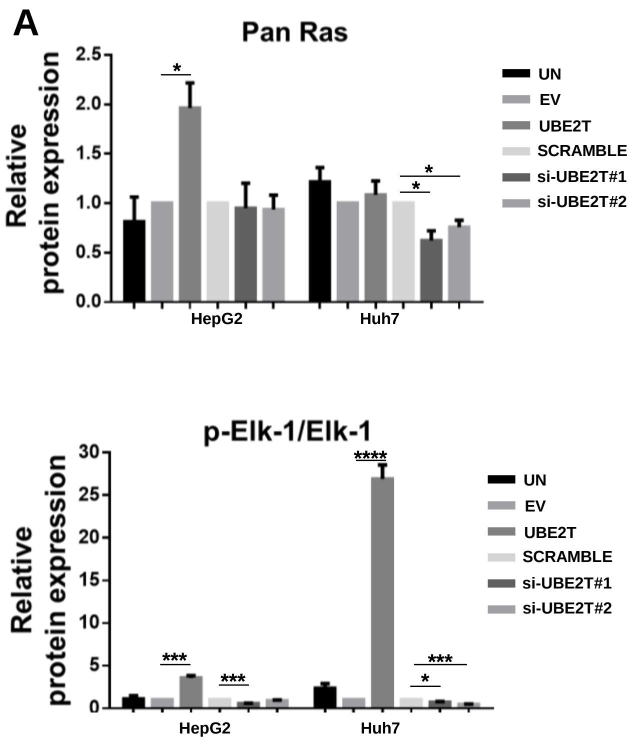
A
Pan Ras
Relative protein expression
2.5
2.0
1.5
1.0
0.5
0.0
*
*
*
HepG2
Huh7
UN
EV
UBE2T
SCRAMBLE
si-UBE2T#1
si-UBE2T#2
c-Myc
Relative protein expression
14
12
10
8
6
4
2
0
*
****
*
****
**
*
HepG2
Huh7
UN
EV
UBE2T
SCRAMBLE
si-UBE2T#1
si-UBE2T#2
p-Elk-1/Elk-1
Relative protein expression
30
25
20
15
10
5
0
****
***
***
***
*
HepG2
Huh7
UN
EV
UBE2T
SCRAMBLE
si-UBE2T#1
si-UBE2T#2

**Figure S4.**

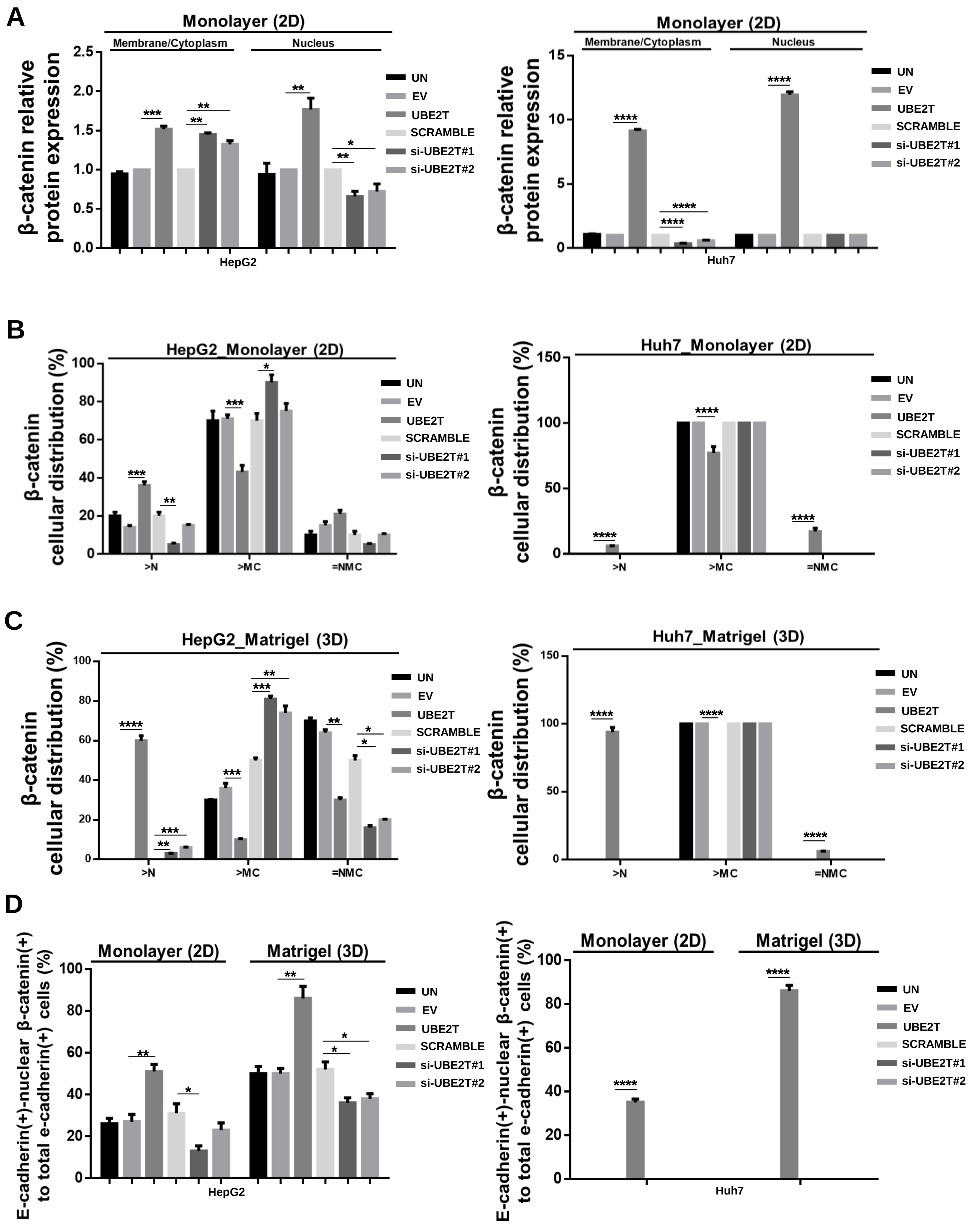

A
Monolayer (2D)
Membrane/Cytoplasm
Nucleus
β-catenin relative protein expression
2.5
2.0
1.5
1.0
0.5
0.0
HepG2
15
10
5
0
Huh7
UN
EV
UBE2T
SCRAMBLE
si-UBE2T#1
si-UBE2T#2
B
HepG2_Monolayer (2D)
Huh7_Monolayer (2D)
β-catenin cellular distribution (%)
100
80
60
40
20
0
150
50
>N
>MC
=NMC
C
HepG2_Matrigel (3D)
Huh7_Matrigel (3D)
D
Monolayer (2D)
Matrigel (3D)
E-cadherin(+)-nuclear β-catenin(+) to total e-cadherin(+) cells (%)
HepG2
Huh7

# Figure S5.

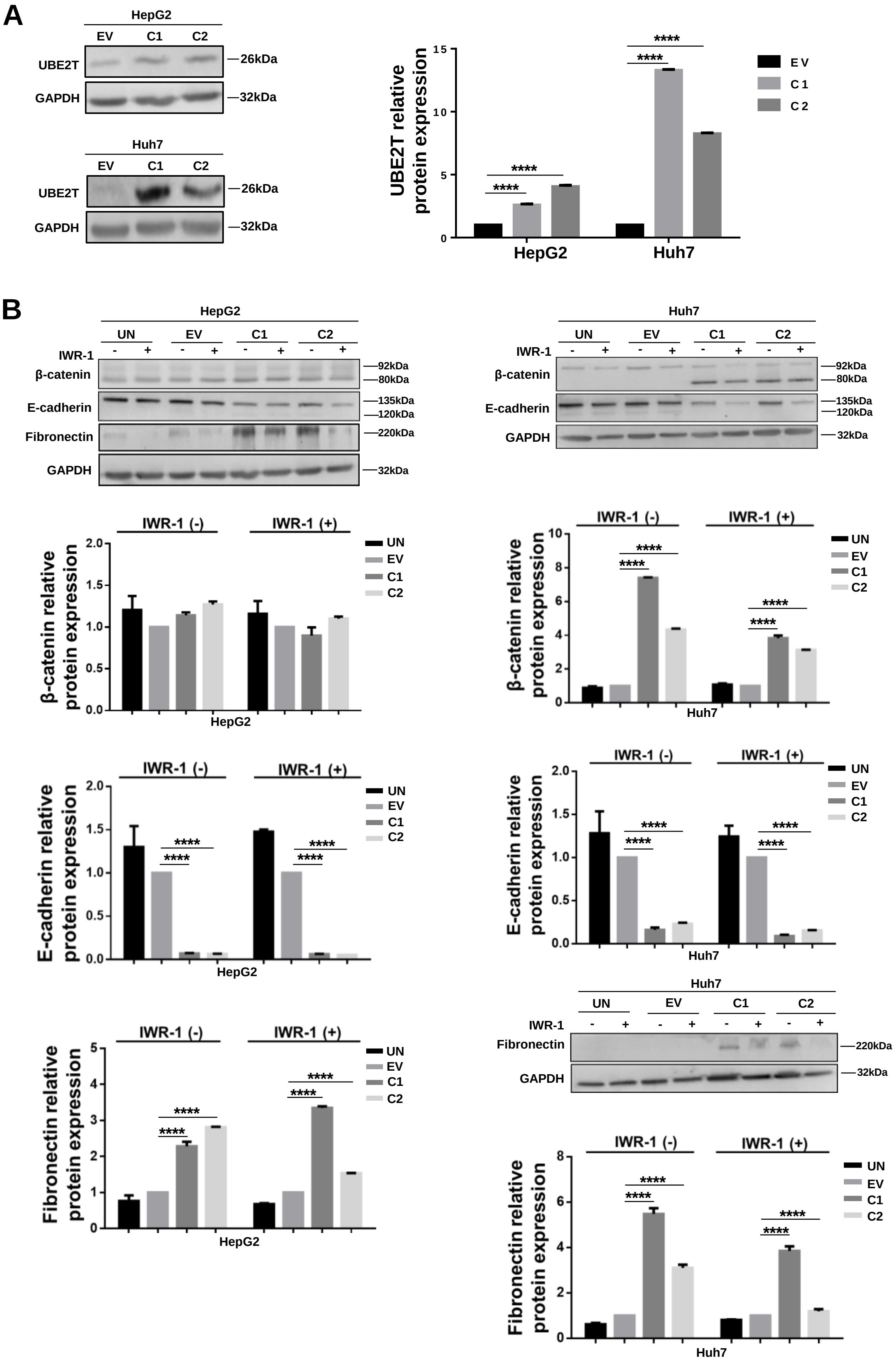
A
HepG2
EV
C1
C2
UBE2T
GAPDH
26kDa
32kDa
Huh7
UBE2T relative protein expression
HepG2
Huh7
****
B
β-catenin
E-cadherin
Fibronectin
IWR-1
UN
92kDa
80kDa
135kDa
120kDa
220kDa
IWR-1 (-)
IWR-1 (+)
β-catenin relative protein expression
E-cadherin relative protein expression
Fibronectin relative protein expression

**Figure S5.**

**C**

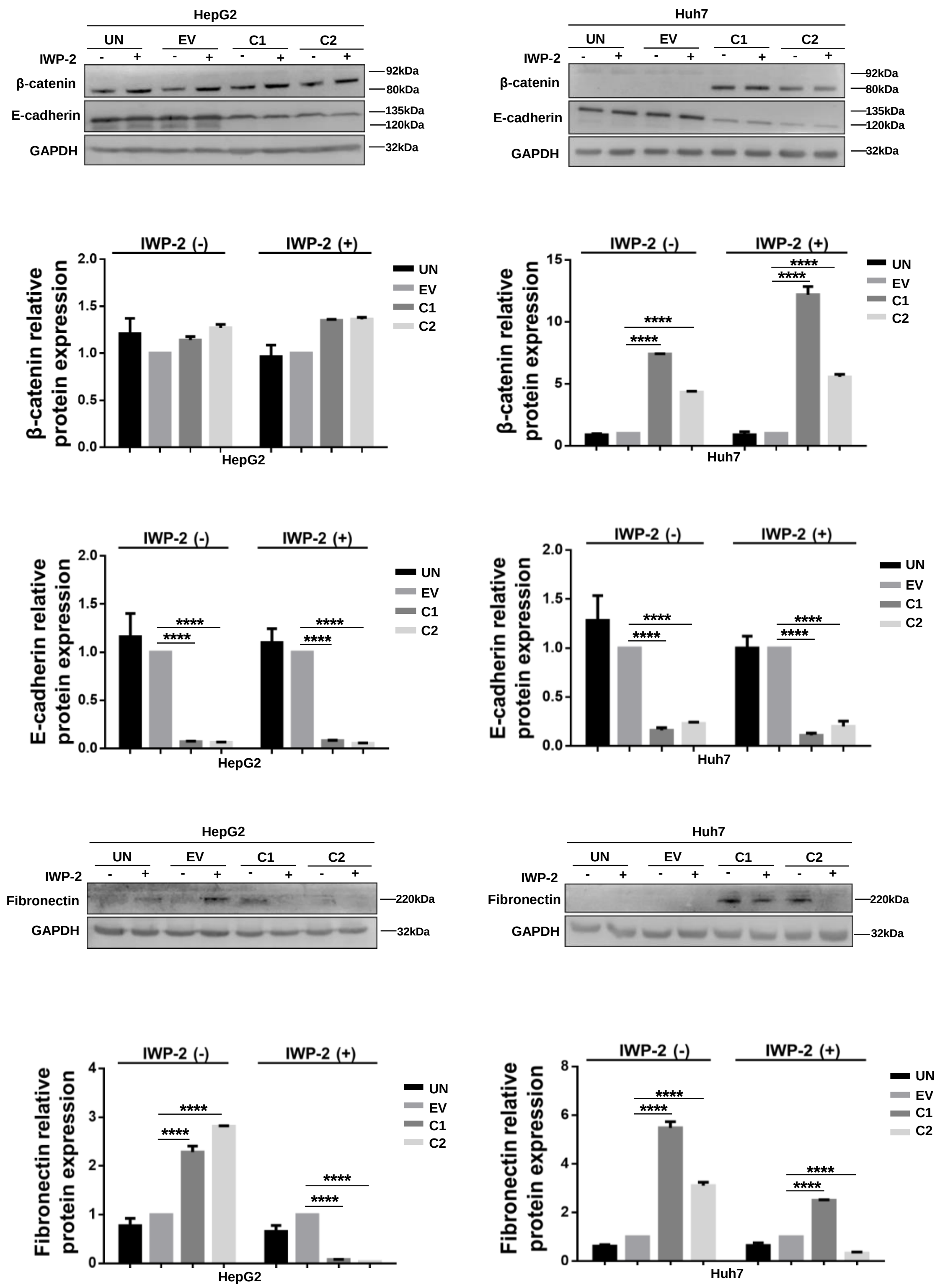
HepG2
UN
EV
C1
C2
IWP-2
β-catenin
E-cadherin
GAPDH
92kDa
80kDa
135kDa
120kDa
32kDa
Huh7
IWP-2 (-)
IWP-2 (+)
β-catenin relative protein expression
E-cadherin relative protein expression
Fibronectin relative protein expression
****
Fibronectin
220kDa

# Figure S5.

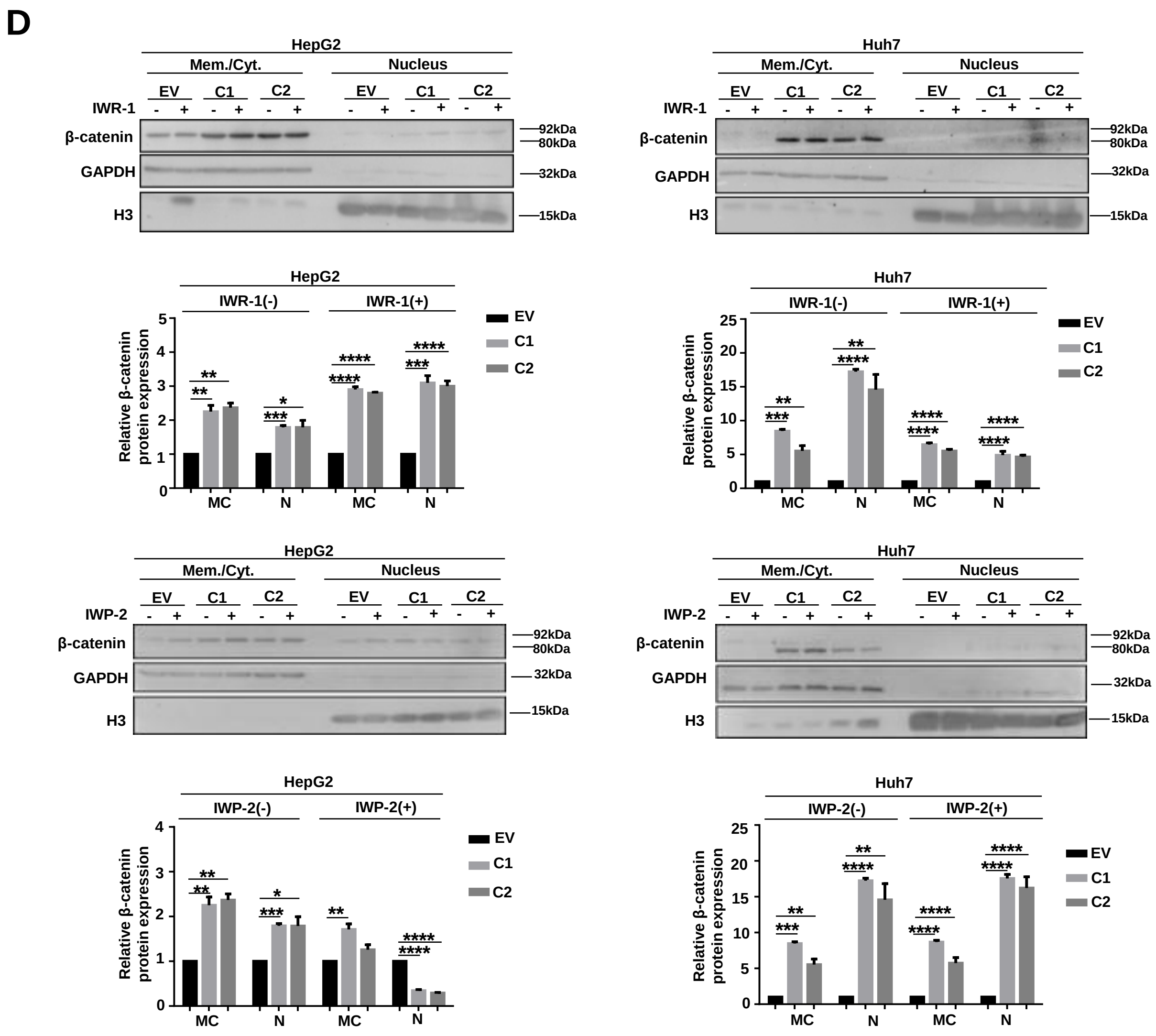
D
HepG2
Mem./Cyt.
Nucleus
EV
C1
C2
IWR-1
β-catenin
GAPDH
H3
92kDa
80kDa
32kDa
15kDa
Huh7
Relative β-catenin protein expression
IWR-1(-)
IWR-1(+)
MC
N
EV
C1
C2
IWP-2
IWP-2(-)
IWP-2(+)

# Figure S5.

**E**

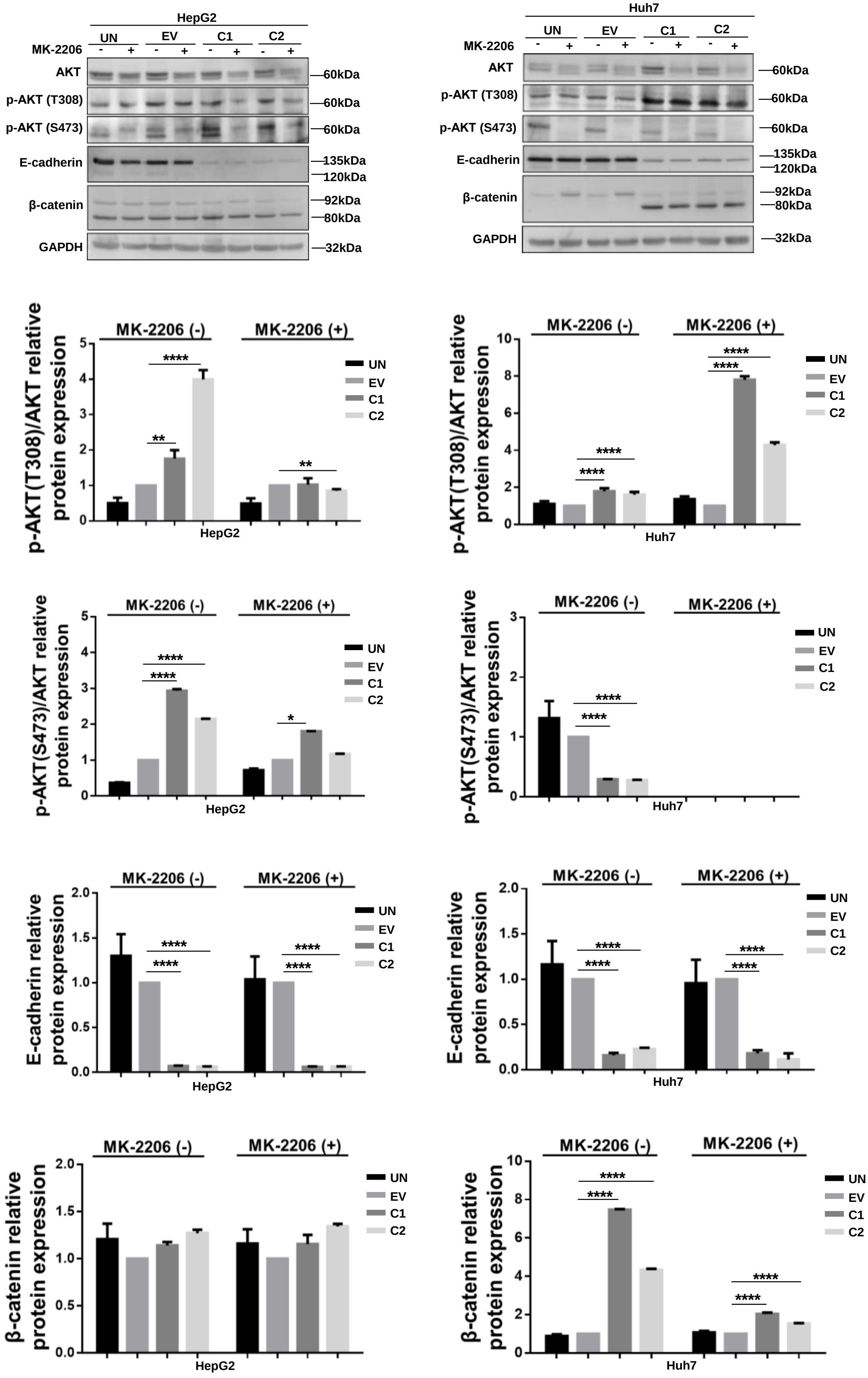
HepG2
UN
EV
C1
C2
MK-2206
AKT
p-AKT (T308)
p-AKT (S473)
E-cadherin
β-catenin
GAPDH
60kDa
135kDa
120kDa
92kDa
80kDa
32kDa
Huh7
MK-2206 (-)
MK-2206 (+)
p-AKT(T308)/AKT relative protein expression
p-AKT(S473)/AKT relative protein expression
E-cadherin relative protein expression
β-catenin relative protein expression

**Figure S5.**

E

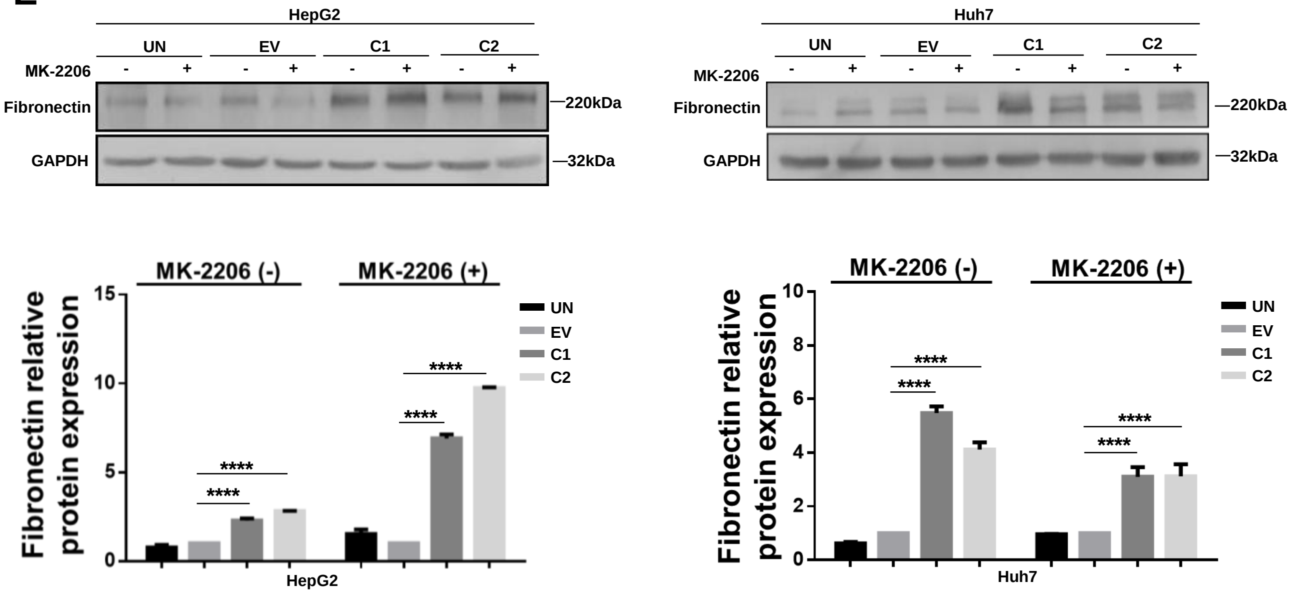
HepG2
UN
EV
C1
C2
MK-2206
Fibronectin
GAPDH
220kDa
32kDa
Huh7
MK-2206 (-)
MK-2206 (+)
Fibronectin relative protein expression
UN
EV
C1
C2
****
HepG2
Huh7

F

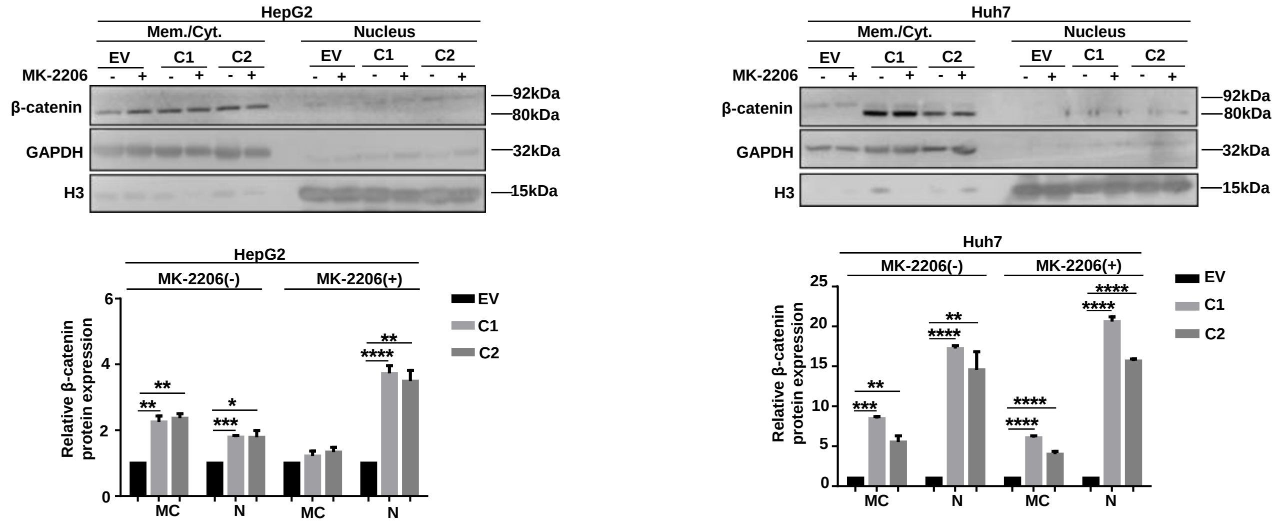
HepG2
Huh7
Mem./Cyt.
Nucleus
EV
C1
C2
MK-2206
β-catenin
GAPDH
H3
92kDa
80kDa
32kDa
15kDa
MK-2206(-)
MK-2206(+)
Relative β-catenin protein expression
MC
N
*
**
***
****

# Figure S6.

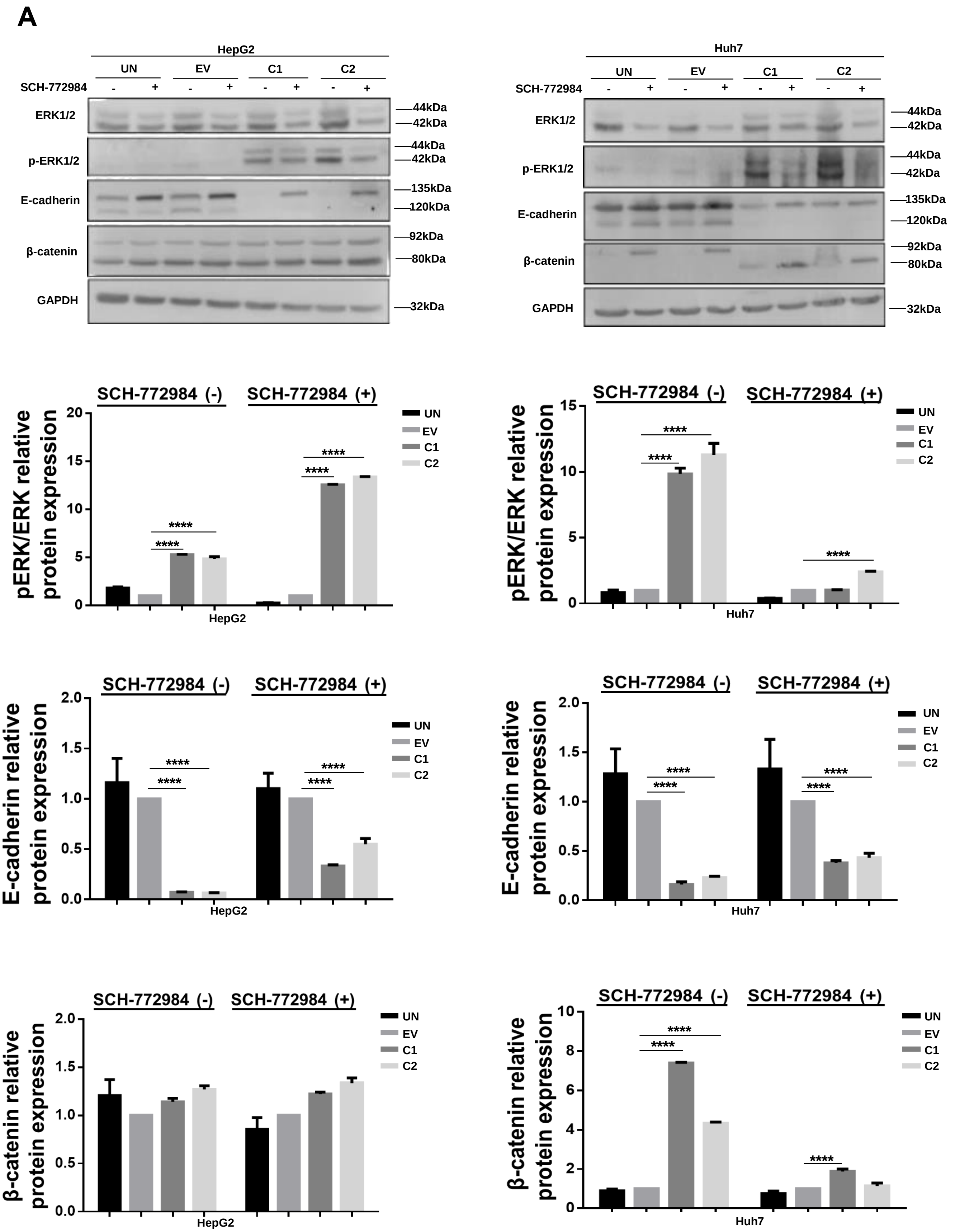
A
HepG2
Huh7
UN
EV
C1
C2
SCH-772984
ERK1/2
p-ERK1/2
E-cadherin
β-catenin
GAPDH
44kDa
42kDa
135kDa
120kDa
92kDa
80kDa
32kDa
SCH-772984 (-)
SCH-772984 (+)
pERK/ERK relative protein expression
E-cadherin relative protein expression
β-catenin relative protein expression
****

**Figure S6.**

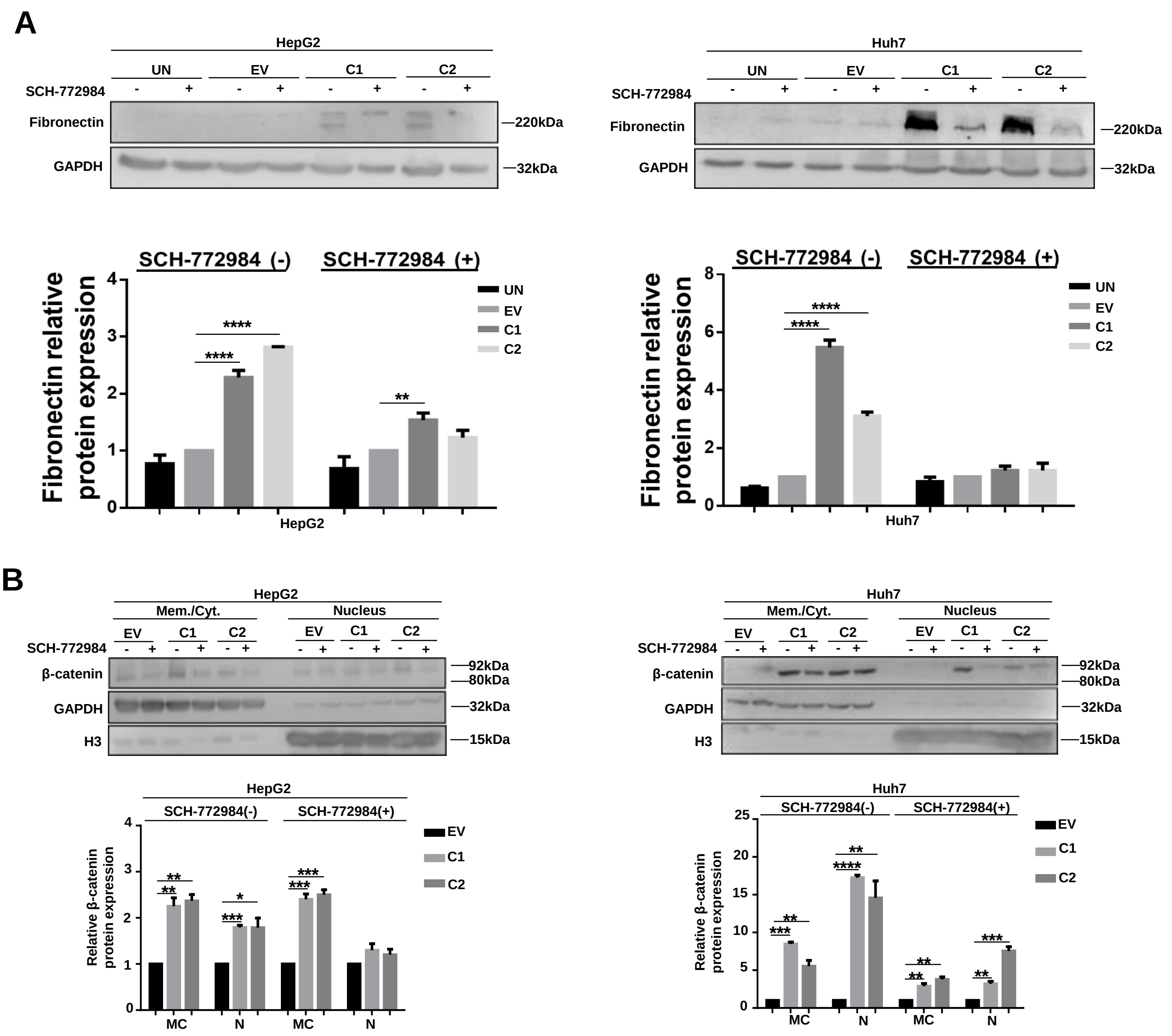
A
HepG2
UN
EV
C1
C2
SCH-772984
Fibronectin
GAPDH
—220kDa
—32kDa
Huh7
SCH-772984 (-)
SCH-772984 (+)
Fibronectin relative protein expression
UN
EV
C1
C2
****
**
HepG2
Huh7
B
HepG2
Mem./Cyt.
Nucleus
β-catenin
GAPDH
H3
92kDa
80kDa
32kDa
15kDa
Huh7
SCH-772984(-)
SCH-772984(+)
Relative β-catenin protein expression
EV
C1
C2
MC
N
*
**
***
****
